# Feature-aware attention convolutional neural network for efficient pulmonary fibrosis classification from chest CT images with FPGA deployment

**DOI:** 10.3389/fmed.2026.1964909

**Published:** 2026-09-30

**Authors:** K. Mahapackialakshmi, G. Jaffino

**Affiliations:** 1 School of Electronics Engineering, Vellore Institute of Technology, Vellore, Tamil Nadu, India

**Keywords:** binary classification, embedded AI, FAA-CNN, FPGA, hardware acceleration, medical imaging, PYNQ-z2

## Abstract

**Introduction:**

Pulmonary Fibrosis (PF) is a progressive interstitial lung disease that requires accurate analysis of chest Computed Tomography (CT) images, while conventional image interpretation is time-consuming and dependent on expert assessment. Although deep learning has demonstrated strong performance for automated PF classification, many existing approaches are primarily designed for software-based execution and do not explicitly consider deployment on resource-constrained embedded hardware.

**Methods:**

To address this limitation, this study proposes a lightweight Feature-Aware Attention Convolutional Neural Network (FAA-CNN) for binary classification of pulmonary fibrosis and normal CT images with FPGA-oriented design considerations. The proposed architecture integrates multi-level feature fusion and feature-wise attention gating to enhance discriminative feature representation while maintaining a compact computational structure. The trained model is translated into synthesizable VHDL using MATLAB HDL Coder and implemented for the PYNQ-Z2 FPGA with a Zynq-7020 device using Xilinx Vivado.

**Results and Discussion:**

Experimental evaluation on 5,120 CT images demonstrates that the proposed FAA-CNN achieves 97.82% accuracy, 97.18% sensitivity, and 98.46% specificity, outperforming the analytical baseline and base CNN. The FPGA implementation achieves timing closure at 47 MHz and utilizes 5,236 LUTs, 4,931 flip-flops, 106.5 BRAM tiles, and 10 DSP48E1 slices. Vivado estimates a total on-chip power of 0.139 W, comprising 0.028 W dynamic and 0.112 W device static power. These results demonstrate the feasibility of combining pulmonary fibrosis image classification with FPGA-oriented implementation.

## 1 Introduction

The significance of Computed Tomography imaging in the current medical diagnosis system is that it offers high-resolution cross-sectional images of the internal body structures. Contrary to traditional x-ray imaging, CT scans generate detailed volumetric images that allow clinicians to detect subtle changes in the pathology of the tissues and thus are also useful in the diagnosis of pulmonary and thoracic diseases. CT imaging has remained popular in identifying abnormalities (fibrosis, tumors, infections, lesions, and structural deformities) to facilitate early and precise clinical intervention. The contrast resolution in CT is the high contrast resolution that allows a detailed analysis of the lung tissues, airways and parenchymal regions and leads to observation of the subtle disease patterns that cannot be observed using conventional radiography. However Manual CT image interpretation is time-consuming, very reliant on radiological skills and prone to inter-observer variability. As the volume of medical imaging data is growing rapidly, the demand of automated and dependable systems of diagnostic assists increases.

The recent developments in AI and deep learning, specifically convolutional neural networks, have improved medical image analysis significantly as there is high performance with regard to classification, segmentation, and detection tasks. Nonetheless, the majority of applications are software-based and rely on GPUs or cloud servers which present issues of high latency, power consumption, cost of hardware, and dependence on connectivity rendering them unsuitable for performance, embedded or resource-constrained clinical settings. Algorithms are nowadays prioritized in current systems, but hardware viability, integration of the system, and deterministic behavior are often neglected, causing a divide between research-level AI systems and usable medical devices. This absence of hardware-friendly design approaches restricts translation to implementable healthcare applications, particularly to point-of-care, remote, and portable diagnostic hardware and systems, which require compact, low-energy, and robust systems.

An attractive way to fill this gap is available in Field Programmable Gate Arrays (FPGAs) where hardware-level acceleration of neural network models can be offered with fine-grained parallelism, deterministic execution and energy-efficient computation. Neural network Inference can be performed in a custom hardware architecture, which is made possible by FPGA-based systems, allowing optimized dataflow, lower memory access latency and processing. The features of FPGAs ensure that they are very suitable in embedded medical AI systems, especially where power efficiency, resource efficiency, and reliability are important. Based on these issues, this paper is motivated by research and development of a fully embedded AI system to classify CT images with the FPGA hardware acceleration. The proposed system is contrasted with software-only solutions in that the design of the algorithm, modelling of hardware, code generation of HDL, and deployment with an FPGA are combined into one implemented system. A proposed model is created for binary classification and the model is converted to hardware via MATLAB HDL Coder to produce synthesizable VHDL code. The resulting hardware design is imported into the Vivado environment, upon which it is synthesized, implemented, and a bitstream is generated. The system is implemented on a PYNQ-Z2 FPGA board, where the classifications were performed, and validation of output is demonstrated on a set of Light Emitting Diodes (LEDs) on the board.

Although deep learning has significantly improved pulmonary fibrosis classification from CT images, most existing methods are designed for GPU-based execution and primarily emphasize classification accuracy without considering hardware-efficient deployment. Similarly, existing FPGA implementations mainly focus on generic CNN acceleration and seldom address pulmonary fibrosis-specific lightweight architectures that balance classification performance, resource utilization, and embedded execution. Consequently, there remains a need for an FPGA-oriented CNN architecture that simultaneously achieves high diagnostic accuracy, low hardware complexity, and efficient implementation for embedded medical imaging applications.

The main contributions of this paper are described as follows:

- A lightweight FPGA-oriented Feature-Aware Attention CNN (FAA-CNN) is proposed by integrating feature fusion and attention mechanisms for efficient pulmonary fibrosis classification from CT images.
- An end-to-end hardware–software co-design framework is developed to translate the proposed FAA-CNN into synthesizable VHDL and deploy it on the PYNQ-Z2 FPGA platform for hardware inference.
- A hardware-efficient implementation strategy is presented to achieve low-resource and low-power FPGA deployment suitable for embedded medical imaging applications.
- The proposed framework enables accurate pulmonary fibrosis classification while maintaining efficient utilization of FPGA logic resources and on-chip power.
- Comprehensive experimental validation is performed through software evaluation, FPGA implementation, ROC analysis, feature visualization, and ablation studies to demonstrate the effectiveness of the proposed framework.

The rest of this paper is structured in the following way. Section 2 provides related work. Section 3 gives the proposed CNN architecture and HDL design methodology. Section 4 describes the FPGA architecture. Section 5 details the hardware implementation at the PYNQ-Z2 platform. Section 6 includes the results and performance analysis of the experiment. The results are discussed in Section 7, and a conclusion is also discussed in Section 8.

## 2 Related works

The latest developments in regard to quantitative imaging biomarkers and deep learning have greatly influenced the diagnosis and prognosis of fibrotic lung disease. The AIIB23 competition (MICCAI-2023) emphasized the significance of airway-informed biomarkers, and loss functions, voxel-wise weighted union and continuity loss were novel, which led to better airway remodelling in complex honeycombing patterns, and eventually demonstrated a potent airway-derived biomarker that outperformed clinical and AI-based measurements of survival prediction by Nan Y et al. (1). Equally, Maddali et al. (2) reported the deep learning CNN algorithm trained on more than 2,000 ILD cases with robust performance (AUC: 0.87) in distinguishing Idiopathic Pulmonary Fibrosis (IPF) against other ILDs using CT scans alone, in which they noted consistent generalization across scanner types and slice thickness and have the potential to eliminate the need to open tissues with invasive biopsies.

In this study, Thillai et al. (3) suggested automated segmentation of CT-based anatomical biomarkers and it was found that the lung, vascular and fibrosis volumes were successfully correlated to the lung function and survival outcomes with longitudinal variations in lung and fibrosis volumes giving the strongest prognostic implications. Taken together, these studies highlight the increased role of automated CT-based biomarkers in the improvement of non-invasive diagnosis, risk stratification, and clinical trial endpoints of IPF. Xu, M et al. (4) have shown that a deep learning model, the Parallel Multi-scale Feature Fusion Network was created to recognize the type of HRCT imaging (UIP, NSIP, OP and normal scans). The model was trained on 130 cases of patients, as well as controls, and was found to be highly diagnostic (92.84) with quick inference (105 s on 18 scans). These results indicate that PMFF Net will be able to offer high-quality decision support that enhances diagnostic consistency and efficiency in clinical practice.

To classify acute exacerbation of idiopathic pulmonary fibrosis AE IPF based on HRCT scans of multi-centre cohorts, a novel 3D deep learning-based algorithm, (SlowFast) was developed. The model had great diagnostic accuracy 93.9 and 86.5 on two test sets and great agreement to radiologist consensus kw up to 0.90 and ROC values of 0.96 and 0.92. Its performance was much better than visual analysis and offered good prognostic information, which points to its possibility of being a good clinical aid to diagnose AE IPF and predict its progression in Huang, X et al. (5). According to Zhang et al. (6), the quantitative features of the HRCT produced were objective indicators of IPF that were linked to the decline in pulmonary function. It has been demonstrated in other research studies that automated segmentation and texture-based analysis the reproducibility is enhanced in comparison to radiologist scoring. Nevertheless, CT metric integration with PFTs is still in its infancy, and combined methods are worth considering in terms of disease diagnosis and progression evaluation.

Jingping Zhang et al. (7) created and tested a deep-learning model to predict imaging phenotypes of IIM-ILD on HRCT scans with good results (AUCs of 0.885 and 0.835, respectively). Their study revealed the helpfulness of class activation mapping and label smoothing methods to improve interpretability and diagnostic quality. The increasing role of AI-mediated quantitative imaging in aiding radiological evaluation of complicated interstitial lung diseases is indicated. A comparative study of vision transformer architecture to detect pulmonary fibrosis through CT scans was suggested by Waseem Sabir et al. (8). Their optimized ViT model had an almost flawless performance where the test accuracy was 100 percent and they scored perfect points in the sensitivity, specificity, F1-score, Matthews Correlation Coefficient (MCC), and Receiver Operating Characteristic (ROC)-AUC (Area Under the Curve) measures. The article shows that deep learning methods based on transformers can be effective diagnostic tools in automated PF classification.

The article by de la Orden Kett Morais et al. (9) summarized the role of deep learning in the imaging of fibrotic lung disease, noting that in addition to its ability to eliminate the limitations of radiologist assessment and quantitative CT, it has the disadvantage of algorithm bias, explainability, and ethical issues. Taken together, these articles and research highlight the rising role of quantitative imaging and AI-based approaches in enhancing the accuracy of diagnosis and monitoring of diseases in fibrotic lung diseases. Wu et al. (10) proposed the feature adaptive transformer network FAT-Net, which is a structure incorporating transformer branches and a CNN-based encoder. They have also designed their way to incorporate a memory-efficient decoder and a feature adaptation module, which makes more efficient use of memory and allows features found on both branches to be successfully combined. Prabin Jose et al. (11) have presented an FPGA-based epileptic seizure detector system based on the EEG signal. Firewall FIR bandpass filtering was used to process the signals and classification was carried out on Zynq-7000 platform using an Extreme Learning Machine (ELM). The difference between normal and seizure patterns could be made using the statistical characteristics that were calculated in sub-bands, and the efficient hardware implementation was enabled by exploiting the use of fast multipliers and LUTs.

Recently, lightweight convolutional networks and attention-based architectures have attracted considerable interest because they offer a favorable balance between feature representation and computational complexity. However, most of these models are optimized for GPU-based execution and require substantial memory bandwidth and computational resources, limiting their deployment on low-cost FPGA platforms. Moreover, existing FPGA implementations mainly focus on accelerating generic CNN inference rather than designing disease-specific hardware-aware architectures for pulmonary fibrosis classification. These limitations motivate the development of an FPGA-oriented lightweight CNN that integrates feature fusion and attention while maintaining low hardware complexity suitable for embedded medical imaging applications.

To position the proposed work within both the pulmonary fibrosis classification and embedded medical-AI domains, Table 1 provides a comparative overview of representative medical imaging approaches and hardware-based implementations. The pulmonary fibrosis studies summarize existing image-based classification capabilities, whereas the FPGA-based studies provide a hardware-deployment perspective in terms of implementation platform and reported hardware-performance characteristics. The proposed FAA-CNN is included to demonstrate its position at the intersection of these two research directions, namely pulmonary fibrosis classification and hardware-oriented embedded inference. The comparison is therefore intended to identify the methodological and deployment gap rather than to imply direct clinical or hardware equivalence among all studies.

**Table 1:** Comparison of existing approaches.

References/Year	Method	Reported performance	Limitations	Hardware deployment
Christe et al. (12)/2019	Custom CNN; UIP vs. non-UIP in HRCT	92% accuracy;	Limited dataset (*n* = 100); overfitting risk	NR
Mishra et al. (13)/2021	CNN (VGG-like); COVID vs. normal CT	97% sensitivity;	Pandemic-specific data; temporal drift	NR
Wong et al. (14)/2021	Custom Fibrosis-Net; fibrosis extent in CT	91% Dice	HRCT-only; FVC prediction indirect	NR
Choi et al. (15)/2022	SOFIA CNN; indeterminate UIP in HRCT	89% accuracy	Binary-only; clinician dependency	NR
Mohan et al. (16)/2022	MIDNet-18; binary lung disease vs. normal	95% F1	Class imbalance; limited to 2 classes	NR
Teramoto et al. (17)/2022	CNN; IPF vs. non-IPF pathology-CT	90% specificity	Histology-dependent; small cohort	NR
Xu et al. (4)/2025	PMFF-Net; UIP/NSIP/OP vs. normal CT	92% precision	Multi-pattern overlap; limited data	NR
Waseem Sabir et al. (8)/2023	ViT-CNN hybrid; fibrosis vs. normal CT	95% AUC	Transformer compute-heavy	NR
Hernández-Trinidad et al. (18)/2024	VGG16 CNN + filters; fibrosis vs. non-fibrosis CT	>97% accuracy	Local dataset (Mexico); post-COVID specific	NR
Hammad et al. (19)/2025	Custom CNN + XAI; malignant vs. benign nodules	96% AUC	Single-center data; nodule size bias	NR
Zhang et al. (7)/2025	DL CNN; ILD fibrosis patterns in HRCT	88% accuracy	Rare disease scarcity; myopathy bias	NR

NR, Not Reported.

As shown in Table 1, existing pulmonary fibrosis classification studies predominantly emphasize software-based model performance, with limited consideration of hardware-oriented deployment. To further examine the hardware-deployment perspective, representative FPGA-based medical AI implementations are summarized separately in Table 1a. This two-level comparison distinguishes the clinical image-classification literature from the broader hardware-acceleration literature and helps position the proposed FAA-CNN at the intersection of pulmonary fibrosis classification and resource-efficient FPGA deployment.

**Table 1a:** Comparison of representative FPGA-based implementations.

References/Year	Application	Method	Hardware deployment	Precision	FF	LUT	Power
A. V et al. (20)	Parkinson's disease detection from hand-drawn circles	Lightweight CNN	Xilinx Zynq UltraScale + ZCU106	Q4.12	741	924	3.178 W,
Proposed FAA-CNN	Pulmonary fibrosis classification from CT	FAA-CNN	PYNQ-Z2/Zynq-7020	16-bit fixed-point	4,931	5,236	0.139 W

Recent hybrid architectures combining convolutional neural networks with Transformer or Mamba-based components have also been explored in medical image classification and disease detection. Mamba is a selective state-space model (SSM) designed to efficiently model long-range dependencies in sequential data (21). However, the primary objective of the present study is to develop a lightweight CNN architecture specifically oriented toward FPGA implementation under resource and computational constraints. CNN–Transformer models typically incorporate attention and token-mixing operations that introduce additional computational and memory requirements, while CNN–Mamba architectures employ state-space-based sequence modeling components that require specialized hardware mapping and optimization. Direct implementation and benchmarking of these architectures on the target PYNQ-Z2 platform would therefore require a separate hardware optimization and evaluation framework. Accordingly, CNN–Transformer and CNN–Mamba architectures were not included as primary experimental baselines in the present study. Their exclusion reflects the hardware-oriented scope of this work rather than an assumption that these architectures provide inferior classification performance.

Table 1a demonstrates that FPGA-based medical AI implementations have been explored for various biomedical applications, confirming the feasibility of hardware-accelerated inference in resource-constrained environments. However, these implementations primarily address applications other than pulmonary fibrosis CT classification. The proposed FAA-CNN extends this hardware-oriented direction to pulmonary fibrosis by combining lightweight feature extraction, feature fusion, and feature-wise attention with FPGA deployment on the PYNQ-Z2 platform.

Despite significant progress in pulmonary fibrosis detection and classification, several limitations remain in existing research. Most of the studies use single-center retrospective datasets that are not very diverse, which limits the generalizability of the model in other clinical contexts. Other issues are inter-observer variability, dependence on non-contrast CT images, and the lack of external validation samples. Class imbalance and small datasets may lead to overfitting, while computationally intensive deep learning models restrict scalability and FPGA deployment in resource-constrained environments. Further, many existing methods are black box models which are not easily interpretable. These shortcomings demonstrate the importance of effective, clear, and optimized hardware-friendly solutions that can be implemented in practice.

Existing studies primarily focus either on improving pulmonary fibrosis classification accuracy using computationally intensive deep learning models or on generic FPGA acceleration for neural network inference. However, very few studies integrate disease-specific lightweight CNN design with hardware-aware optimization for efficient FPGA deployment. Consequently, there remains a need for an FPGA-oriented CNN architecture that simultaneously achieves accurate pulmonary fibrosis classification, low computational complexity, efficient resource utilization, and embedded implementation. To address this gap, this work proposes a lightweight Feature-Aware Attention CNN (FAA-CNN) integrated with an end-to-end hardware–software co-design framework for FPGA deployment on the PYNQ-Z2 platform.

## 3 Proposed system architecture

The proposed system adopts an end-to-end hardware–software co-design framework for pulmonary fibrosis classification by integrating a lightweight Feature-Aware Attention CNN (FAA-CNN) with FPGA-based embedded implementation. Unlike conventional AI pipelines that prioritize software execution, the proposed framework is designed with hardware-aware considerations, including computational efficiency, low resource utilization, and sequential FPGA inference. The overall workflow comprises CT image preprocessing, feature extraction using the proposed FAA-CNN, feature fusion and attention-based enhancement, binary classification, automatic HDL generation, FPGA synthesis, and on-board hardware validation on the PYNQ-Z2 platform. This unified framework enables efficient translation of the trained CNN into synthesizable hardware while supporting low-power medical image classification.

### 3.1 Overall pipeline

Figure 1 illustrates the complete end-to-end workflow of the proposed embedded AI framework for pulmonary fibrosis classification. Initially, lung CT images are acquired and preprocessed through image resizing and intensity normalization to generate standardized inputs for the proposed Feature-Aware Attention CNN (FAA-CNN). The FAA-CNN performs hierarchical feature extraction using lightweight convolutional blocks, feature fusion, and attention-guided feature refinement to improve discrimination between normal and pulmonary fibrosis CT images while maintaining computational efficiency for FPGA deployment. Following software validation in MATLAB, the trained network is automatically translated into synthesizable VHDL using MATLAB HDL Coder. The generated hardware description is synthesized and implemented in the Xilinx Vivado Design Suite to produce the FPGA bitstream for deployment on the PYNQ-Z2 platform. Finally, sequential FPGA inference is verified through on-board hardware execution, where the classification results are displayed using the FPGA LEDs. This integrated hardware–software pipeline enables efficient, low-power, and pulmonary fibrosis classification suitable for embedded medical imaging applications.

**Figure 1:**
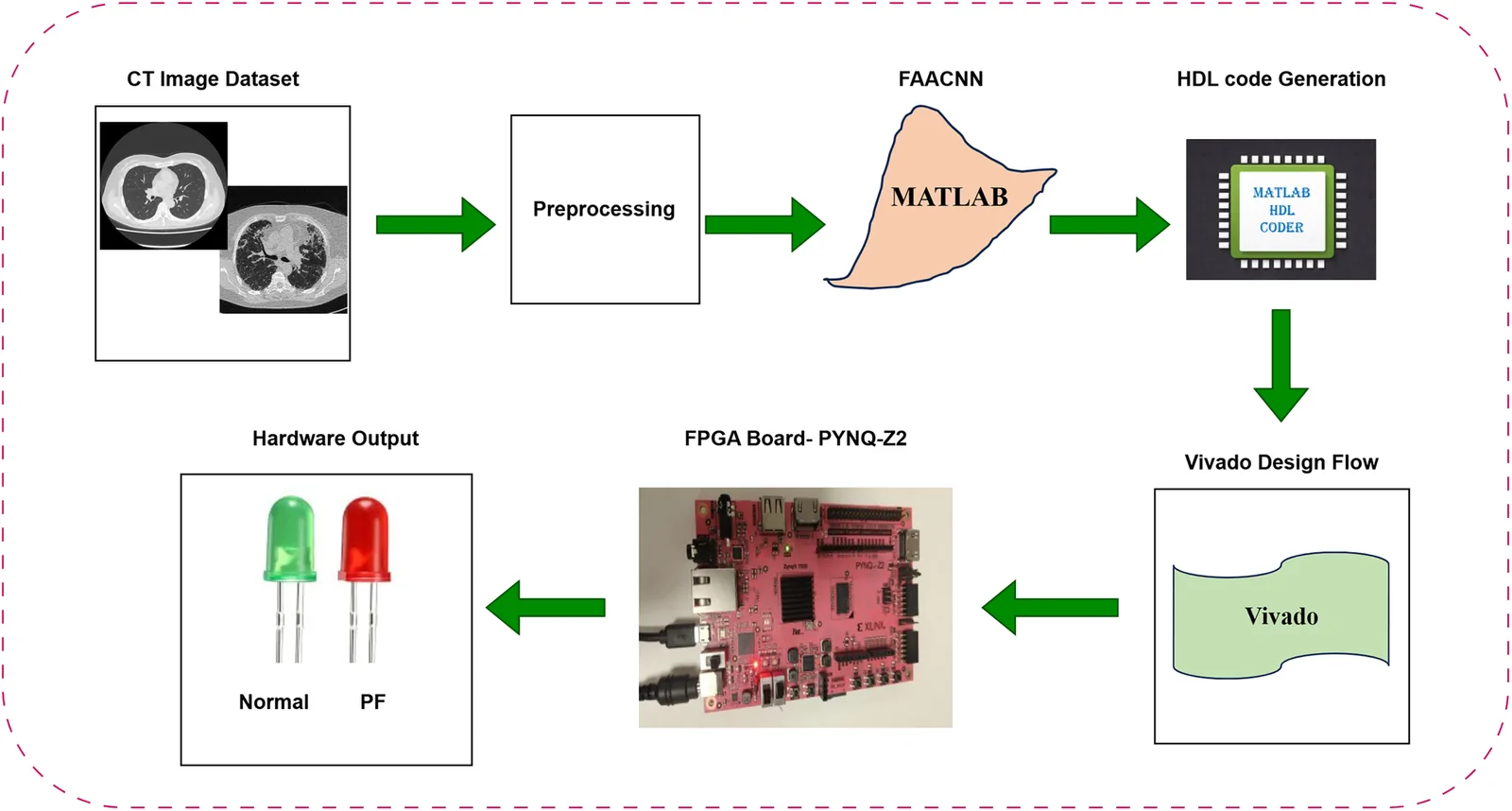
An embedded AI framework for FPGA-based CNN classification of CT images.

### 3.2 Proposed CNN architecture

The original CNN is a lightweight serial architecture of binary classification of CT scans as either Normal or Pulmonary Fibrosis. The 128 × 128 CT image is then fed through three convolutional blocks consisting of 3 × 3 kernels with increasing filter depths (8, 16, 32) followed by LeakyReLU activation to extract low-level edge-based features, interstitial texture patterns, and high-level global lung structures in that order. Global average pooling is adopted instead of feature flattening to reduce the number of trainable parameters, minimize memory requirements, and simplify FPGA implementation while preserving global discriminative information, and salient information conservation. Lastly, a one-neuron fully connected layer that has a sigmoid activation generates the classification output. This model which is computationally efficient does not explicitly interact with multi-scale features or highlight regions which may restrict its capacity to resolve fine fibrotic structures.

The proposed Feature-Aware Attention Convolutional Neural Network (FAA-CNN) is designed as a lightweight binary classification architecture for pulmonary fibrosis detection from 128 × 128 × 1 CT images. The network begins with three convolutional blocks consisting of 3 × 3 convolutions with 8, 16, and 32 filters, respectively, followed by ReLU activations. To enable fusion of hierarchical representations with different channel dimensions, the low-, mid-, and high-level feature maps are projected to a common 32-channel representation using 1 × 1 convolutional layers. The resulting feature maps are combined through the Feature-Aware Feature Fusion (FAFF) module. In the implemented architecture, the fusion coefficients are fixed to *α*₁ = *α*₂ = *α*₃ = 1; hence, the FAFF performs equal-weight element-wise feature fusion rather than learnable weighted fusion. The fused representation is subsequently processed by a 1 × 1 attention convolution followed by a sigmoid activation to generate a 128 × 128 × 32 attention map. This attention map is multiplied element-wise with the fused feature representation to perform feature gating. Global average pooling reduces the spatial representation to a 32-dimensional feature vector, which is passed to a single-neuron fully connected layer and sigmoid output for binary classification into Normal and Pulmonary Fibrosis classes. The entire design contains 17 layers including the input layer and has 8,865 trainable parameters.

Figure 2 demonstrates the transformation of the baseline into the proposed CNN for binary classification. The baseline CNN adopts a shallow three-stage convolutional architecture to progressively learn hierarchical image representations while maintaining low computational complexity. A 3 × 3 convolution kernel is selected to effectively capture local fibrosis textures with fewer parameters than larger kernels, making it suitable for FPGA implementation. The filter dimensions (8, 16, and 32) are gradually increased to balance feature representation capability and hardware resource utilization. Furthermore, Global Average Pooling (GAP) replaces conventional fully connected feature flattening to significantly reduce trainable parameters, memory consumption, and hardware complexity, thereby improving deployment efficiency on resource-constrained FPGA platforms.

**Figure 2:**
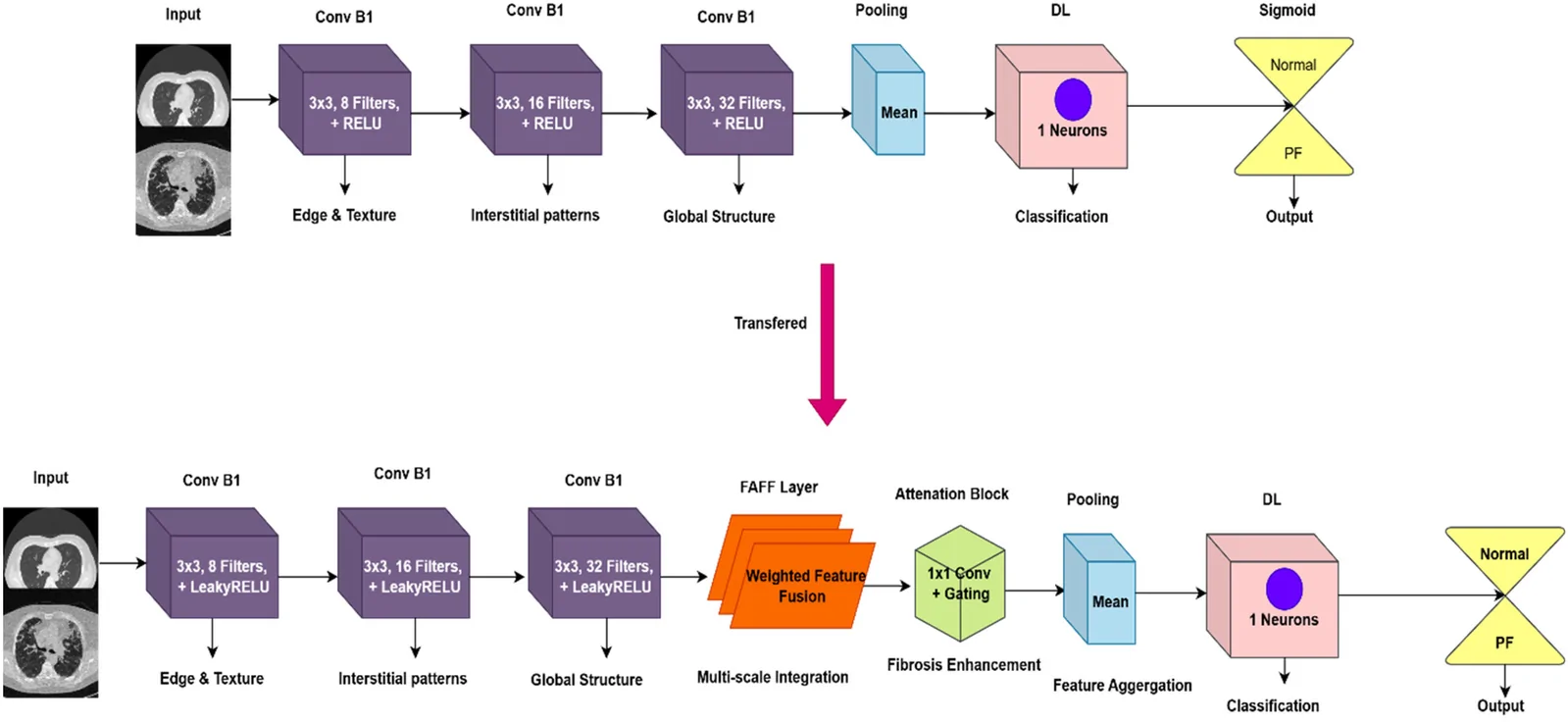
Transformation of the baseline CNN into the proposed CNN for binary CT image classification.

The proposed model extends the baseline CNN by incorporating a Feature-Aware Feature Fusion (FAFF) module and a lightweight attention mechanism to enhance pulmonary fibrosis classification. The resulting architecture, referred to as the Feature-Aware Attention CNN (FAA-CNN), preserves the lightweight characteristics of the baseline while improving discriminative feature representation. The FAFF module adaptively integrates low-level, mid-level, and high-level feature maps extracted from the three convolutional blocks using learnable weighted fusion, thereby preserving both fine-grained texture information and high-level semantic features relevant to pulmonary fibrosis. Unlike conventional feature concatenation or element-wise addition, the proposed weighted fusion strategy emphasizes informative hierarchical representations while minimizing redundant feature propagation, resulting in improved feature utilization with low computational overhead. The mathematical formulation of the proposed FAFF module is presented in the following equations. The attention module utilizes lightweight 1 × 1 convolution and gating operations instead of computationally expensive self-attention mechanisms, thereby improving discriminative feature representation while maintaining low hardware complexity.

The initial convolution operations are identical for both the baseline and proposed CNN. Initially, the input CT image is Equation 1

$$X\in P^{128x128}$$ (1)

The Convolution Blocks are represented with the *l*-th convolution layer, the feature map is computed by Equation 2

$$F_{l}=\sigma (W_{l\;}*F_{l-1}+b_{l}),\;l=\{1,2,3\}$$ (2)

Where * denotes the 2D convolution, $W_{l\;}$ and $b_{l}$ are learnable weights and bias, σ() is the LeakyReLU activation function. Fusion and attention blocks are introduced after the convolution operation to boost performance efficiency. FAFF equations are represented with multi-scale features that are fused using weighted summation in Equation 3

$$F_{\;fus}=\overset{3}{\underset{l=0}{\sum }}\alpha_{l}F_{l}$$ (3)

Where $\alpha_{l}$ is the fusion weights associated with the l-th feature map and $F_{\;fus}$ denotes fused features. In this work, equal fusion weights (*α*₁ = *α*₂ = *α*₃ = 1) are employed to maintain architectural simplicity and FPGA compatibility. In the executed FAA-CNN, the fusion coefficients are set at *α*₁ = *α*₂ = *α*₃ = 1 instead of being acquired through optimization. As a result, the FAFF module executes an equal-weight element-wise summation of the projected low-, mid-, and high-level feature representations. This specific formulation was chosen to diminish hardware intricacy and enhance FPGA deployment. Next, the Attention Module block added feature-wise attention is applied using a 1 × 1 convolution and gating represented in Equations 4 and 5

$$M=\sigma (W_{a\;}*F_{\;fus}+b_{a})$$ (4)

$$F_{at}=M⊙F_{\;fus}$$ (5)

Where, ⊙ is represents the elementwise multiplication. Then, reduce the dimensionality by using a pooling operation, that is, global average pooling. This pooling is calculated by Equation 6,

$$Avg_{k}=\frac{1}{H\times w}\sum_{i=1}^{H}\sum_{\;j=1}^{w}F_{at}^{\left(k\right)}\;(i,j)$$ (6)

The dense layer given in Equation 7,

$$z=W_{d}^{T}Avg+b_{d}$$ (7)

After the dense layer, classification is performed using the binary sigmoid equation shown below Equation 8,

$$C=\frac{1}{1+e^{-z}}$$ (8)

Where c denotes the classification of the diseases.

The proposed FAFF module adaptively combines hierarchical feature maps extracted from the three convolutional blocks through learnable weighted fusion. This enables the network to preserve complementary low-level texture information and high-level semantic representations associated with pulmonary fibrosis. Compared with conventional feature concatenation, the proposed fusion strategy reduces redundant feature propagation while maintaining low computational overhead, thereby improving feature representation and supporting efficient FPGA implementation. The fused feature representation is subsequently refined using a lightweight attention module based on 1 × 1 convolution and gating operations. The attention mechanism selectively enhances fibrosis-relevant regions while suppressing irrelevant background responses, thereby improving discriminative feature learning. The use of 1 × 1 convolution minimizes parameter count and computational complexity while preserving channel-wise feature interactions, making the attention module suitable for resource-constrained FPGA implementations. Unlike transformer-based self-attention mechanisms, the proposed module introduces significantly lower computational and memory complexity, making it well-suited for FPGA deployment. The refined feature maps are then compressed using Global Average Pooling (GAP) and forwarded to a fully connected layer with sigmoid activation for binary classification.

Table 2 shows the design rationale of the proposed FAA-CNN. The integration of hierarchical feature extraction, adaptive feature fusion, and lightweight attention results in a hardware-aware CNN architecture that balances diagnostic accuracy with computational efficiency, making it suitable for FPGA-based embedded medical imaging applications.

**Table 2:** Design rationale of the proposed FAA-CNN.

Module	Purpose	Hardware benefit
Conv Block 1 (8 filters)	Extract low-level edge features	Low computational cost
Conv Block 2 (16 filters)	Learn texture representations	Balanced feature extraction
Conv Block 3 (32 filters)	Capture high-level fibrosis patterns	Improved semantic discrimination
FAFF Module	Adaptive multi-scale feature fusion	Efficient feature reuse with low overhead
Attention Module	Emphasize fibrosis regions	Lightweight enhancement using 1 × 1 convolution
Global Average Pooling	Compress feature maps	Reduces parameters and memory usage
Fully Connected + Sigmoid	Binary classification	Minimal hardware resources

Algorithm 1: FPGA-Oriented Proposed CNN Classification

Input: CT image

Output: Class label (Normal/Pulmonary Fibrosis)

Preprocess input image X (resize, normalize)

F1 ← Conv(X, W1) + b1; Apply LeakyReLU

F2 ← Conv(F1, W2) + b2; Apply LeakyReLU

F3 ← Conv(F2, W3) + b3; Apply LeakyReLU

F_fus_ ← *α*1·F1 + *α*2·F2 + *α*3·F3

M ← sigmoid(Conv1 × 1(F_fus_))

F_at_ ← M ⊙ F_fus_

Avg_k_ ← GlobalAveragePooling(F_at_)

z ← w_d_ · g + b_d_

c ← sigmoid(z)

if c ≥ 0.5 then

Output ← Pulmonary Fibrosis

else

Output ← Normal

end if

A lightweight hardware-aware CNN designed from the beginning for efficient FPGA implementation by combining hierarchical feature extraction, adaptive feature fusion, lightweight attention, and parameter-efficient classification.

### 3.3 HDL code generation

Figure 3 illustrates the HDL-based design flow used to convert the trained CNN into a hardware-accommodating implementation with MATLAB HDL Coder. The process is partitioned into software and hardware domains to illustrate hardware-software co-design. The trained CNN model in MATLAB is analyzed in terms of layer analysis, fixed-point data analysis, and HDL compatibility verification in the software field to confirm its appropriateness in FPGA implementation. MATLAB HDL Coder then automatically produces Register-Transfer Level(RTL) and synthesizable VHDL modules of CNN architecture. Its resultant VHDL gets introduced into the Vivado design software to be synthesized, implemented and generate bitstreams. This is a process that guarantees that the software model and hardware implementation are consistent to achieve dependable FPGA deployment.

**Figure 3:**
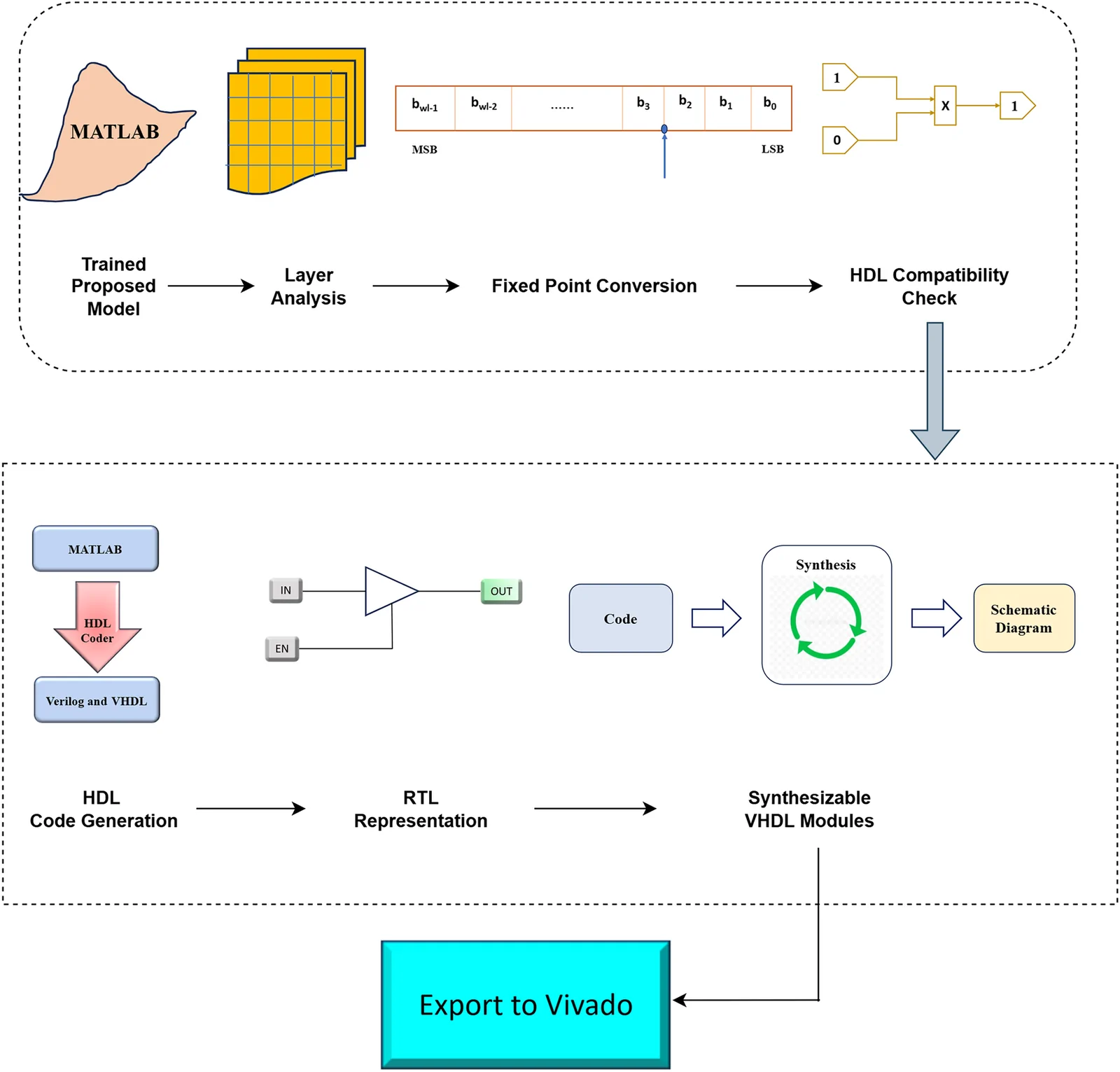
HDL-based design flow for CNN implementation using MATLAB HDL coder.

### 3.4 Threshold-based fibrosis score classifier

To create a hardware-efficient baseline and facilitate FPGA implementation, a threshold-based fibrosis classification algorithm was created through statistical intensity analysis of CT scans. This method, in contrast to convolutional neural networks, is based on a mathematically defined fibrosis score whose computation is based on pixel intensity distribution. First, the size of all CT images was reduced to a standard resolution of 128 × 128 pixels, and to provide consistency and minimise the number of computations. Let I(x,y) represent the intensity of the pixel at the point (x, y), the intensity of a pixel is between 0 and 255. Areas of pulmonary fibrosis are generally observed to be of high-intensity because of tissue scarring and structural anomalies. Thus, candidate fibrosis pixels were identified with the help of a predefined pixel intensity threshold $T_{p}$. The Fibrosis Score(FS) was estimated by dividing the number of pixels with values above this limit by the number of pixels in the image given in Equation 9,

$$FS=\frac{\sum_{\left(x,y\right)}P(I(x,y)>T_{p})}{H\;x\;W}$$ (9)

Where P(.) is the indicator function, $T_{p}\;$ denotes the pixel intensity threshold, and H and W represent the image height and width. This fibrosis score represents the proportion of lung tissue affected by high-intensity abnormalities. The fibrosis score threshold $T_{f}$ was optimized automatically on a grid search to reach the optimum decision boundary. Various threshold values between 0.01 and 0.50 were established and the threshold that produced the best classification accuracy was chosen. The best threshold was determined as,

$$T_{f}=0.44$$

The final classification was obtained from the following conditions given in Equation 10,

$$\mathrm{Outpu}t_{\mathrm{class}}\{\begin{array}{cc} PF,\; & |\mathrm{if}\;FS>T_{f}\;\\ \mathrm{Normal},\; & |\mathrm{otherwise}\; \end{array}$$ (10)

## 4 FPGA hardware architecture

The FPGA hardware architecture is designed to implement the proposed model effectively on reconfigurable hardware while achieving efficient resource utilization through sequential resource sharing. The architecture adopts a modular, hierarchical design, enabling seamless integration of the CNN computational core with FPGA input–output interfaces and on-board control logic. VHDL is used to implement the entire hardware system, which is then synthesized and deployed on the PYNQ-Z2 FPGA platform.

### 4.1 VHDL integration

The HDL code generation stage leads to the VHDL representation of the CNN which is the main computational unit of the FPGA system. The derived VHDL modules are the functional blocks of the CNN, which are convolution blocks, activation blocks, pooling blocks, and classification blocks. They are implemented in RTL constructs which facilitate an efficient mapping to FPGA logic resources. The CNN layers are represented by specific hardware blocks, which have input and output interfaces that are explicitly defined and have controllable data flow and can be executed in pipelines. Fixed-point arithmetic is used all over the design to minimize the complexity of the hardware and enhance the computational speed as much as possible without compromising in classification. The VHDL design has a modular design that allows the scaling of its structure and easy integration with other system components.

### 4.2 Top wrapper design

Figure 4 shows the FPGA hardware architecture of the proposed system, incorporating the CNN processing core with a top-level control wrapper. The CNN core, synthesized as VHDL using MATLAB HDL Coder, performs the binary classification inference. The control wrapper provides clock and reset management and interfaces the CNN core with the FPGA input and output resources. The input image data are supplied to the CNN through the implemented sequential input interface, while the classification result is provided through the output interface and displayed using the onboard LEDs. The architecture separates the CNN computation from the top-level control and I/O interface, enabling integration of the proposed CNN on the PYNQ-Z2 FPGA platform.

**Figure 4:**
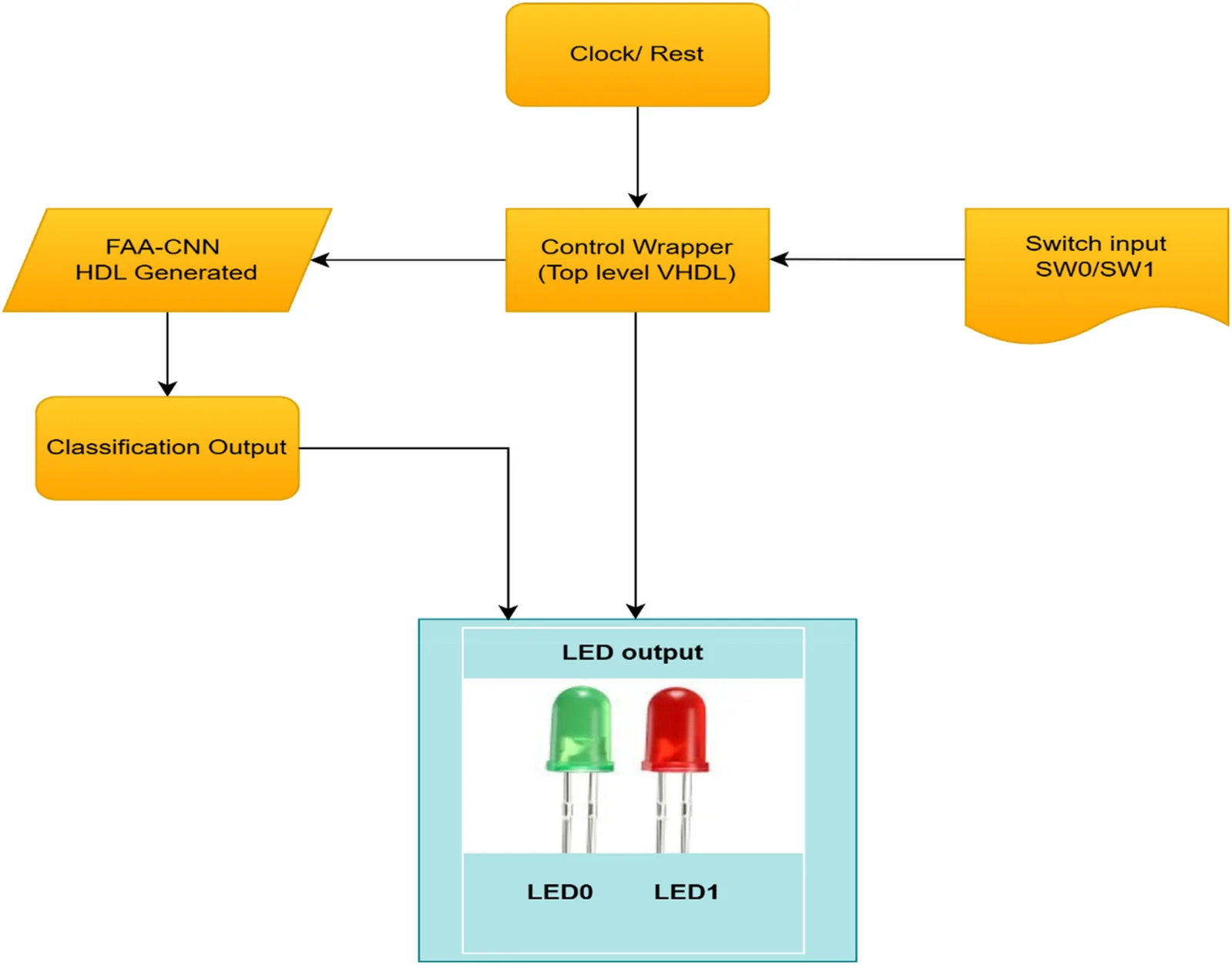
Block diagram of FPGA architecture with CNN core and top-level wrapper integration.

### 4.3 Vivado synthesis and implementation flow

The entire VHDL design, comprising the CNN core and top-level wrapper is imported into the Xilinx Vivado design package to generate hardware and successfully implement it. In the synthesis step, the RTL design is converted into a set of gates which has been optimized to the target FPGA architecture. This is done to guarantee logical correctness, timing viability and the effective utilization of FPGA resources of the form of LookUp Tables (LUTs), Flip-Flops (FFs), Block RAMs(BRAMs) and Digital Signal Processing (DSP) blocks. After the synthesis stage, the implementation stage is done which involves placement and routing of the design to the actual physical FPGA fabric. Timing constraints are implemented in order to have stable operation at the intended clock frequency. The analysis of implementation outcomes is done to confirm the timing closure, resource usage, and routing efficiency. The fact that the CNN architecture works with the limitations of the PYNQ-Z2 FPGA platform has been proved successfully.

### 4.4 Bitstream generation and FPGA configuration

After synthesis and implementation has been passed, a configuration bitstream is created with Vivado. This stream of bits has the low-level configuration information that is needed to program the FPGA with the CNN architecture and control logic designed. The resulting bitstream is available on the PYNQ-Z2 board and loaded on the FPGA, allowing the CNN classifier to be executed at the hardware level. Once configured, the FPGA is a standalone embedded classifier. The CNN process of inference is completely implemented in hardware, and the classification outcomes are produced on the fly. On-board LED indicators represent the decision on the output, directly implying the physical validation of the system functionality. This has proven the accuracy and consistency of the CNN implementation in the FPGA.

### 4.5 Hardware validation strategy

Physical components of a board are used to monitor the functionality of the FPGA hardware architecture by monitoring the system output. The outcome of the binary classification is converted to separate LED outputs with each LED having a particular class. This type of hardware-based validation method does not require use of software debugging systems, and proves that the embedded AI system can work independently. The validation process using physical outputs will be used to make sure that the FPGA hardware produces the classification decisions on its own, which is a guarantee of the effective integration of the CNN and HDL logic as well as the FPGA resources. This validation design shows the feasibility of the architecture to be used in embedded medical systems.

## 5 PYNQ-Z2 hardware implementation

The last phase of the suggested system is to deploy and test the FPGA-based CNN classifier on the PYNQ-Z2 board. This platform is chosen because it is appropriate in embedded AI applications, has a balance between computing ability, low power usage, and simplicity of hardware interaction. The implementation illustrates execution of CNN model on solely FPGA hardware, physical input control and output display.

### 5.1 Board configuration

PYNQ-Z2 board is designed on the Xilinx Zynq-7020 System-on-Chip(SoC), which is a combination of programmable logic(PL) and a processing system(PS). The CNN inference is performed using programmable logic as the main tool in the proposed implementation, which guarantees the acceleration of hardware and deterministic behavior. The resulting FPGA bitstream, which consists of the CNN core and top-level wrapper logic, is loaded into the PYNQ-Z2 board. Clock and reset signals are done in a way that stable system running is maintained and on-board resources are used to initialize the design. After setup, the FPGA would work as an independent classification device and would not require the services of external processors or software execution.

### 5.2 Input interface and HDL verification

The input interface of the proposed FPGA CNN is designed to accept a preprocessed 128 × 128 CT image as a sequential stream of 16-bit pixel samples. The image samples are supplied to the HDL-generated CNN in row-major order using the x_in, valid, and start control signals. The input samples and intermediate feature maps are stored using the on-chip BRAM resources of the FPGA implementation.

For functional verification, the identical preprocessed CT image utilized as input for the MATLAB CNN was provided consecutively to the HDL implementation. The classification results obtained from the HDL-generated CNN have been compared with the equivalent MATLAB outcomes to validate the operational coherence of the FPGA-focused implementation. A binary classification employs a probability threshold of 0.5: a probability beyond 0.5 signifies pulmonary fibrosis, whereas a probability of 0.5 or lower denotes a normal condition.

The FPGA design was verified through HDL simulation and Vivado implementation analysis. The PYNQ-Z2 platform was used as the target FPGA device for synthesis, placement, routing, resource utilization, and timing analysis.

### 5.3 Switch and LED interface

The high-level wrapper offers board-level management and output connections for the FPGA deployment. The accessible control signals facilitate system reset and inference regulation, whereas the classification output is assigned to the LED interface. A probability beyond 0.5 triggers the pulmonary-fibrosis indication, while a probability of 0.5 or lower activates the normal indication. These interfaces offer a straightforward hardware-level method for regulating and monitoring the executed CNN function.

### 5.4 LED output mapping

The binary classification output of the CNN is mapped to two LED output signals in the top-level FPGA wrapper. The two LEDs represent the two classification classes, namely pulmonary fibrosis and normal. A probability value greater than 0.5 corresponds to pulmonary fibrosis, activating the pulmonary-fibrosis LED while the normal LED remains off. Conversely, a probability value less than or equal to 0.5 corresponds to a normal case, activating the normal LED while the pulmonary-fibrosis LED remains off.

The LED interface is implemented as part of the FPGA design to provide a simple hardware-level indication of the binary classification result. The CNN functionality was verified through HDL simulation by comparing the HDL output with the corresponding MATLAB classification result.

### 5.5 Fixed-point representation, quantization, and numerical validation

To enable hardware-efficient implementation of the proposed FAA-CNN on the FPGA, fixed-point arithmetic was employed for the input data, network weights, intermediate feature maps, accumulators, and final output. The fixed-point formats were selected according to the numerical ranges observed from the complete test set of total images. Table 3 summarizes the corresponding word lengths, fractional-bit positions, signedness, and numerical ranges used for each computational stage.

**Table 3:** Fixed-point numerical formats used in the FPGA implementation.

Data/Signal	Signedness	Word length	Fraction length	Fixed-point format
Input	Unsigned	16 bits	15	UQ0.15
LeakyReLU1 feature map	Unsigned	8 bits	7	UQ1.7
LeakyReLU2 feature map	Unsigned	8 bits	7	UQ1.7
LeakyReLU3 feature map	Unsigned	16 bits	11	UQ5.11
Conv1 weights	Signed	16 bits	16	Q0.16
Subsequent weights	Signed	16 bits	15	Q1.15
Biases	Signed	16 bits	17	Q1.17
MAC accumulator	Signed	40 bits	31	Q9.31
Projection-Low	Signed	16 bits	14	Q2.14
Projection-Mid	Signed	16 bits	14	Q2.14
Projection-High	Signed	16 bits	9	Q7.9
FAFF output	Signed	16 bits	8	Q8.8
Attention convolution	Signed	16 bits	7	Q9.7
Attention sigmoid	Unsigned	8 bits	7	UQ1.7
Attention-gated feature map	Signed	16 bits	10	Q6.10
GAP output	Signed	16 bits	12	Q4.12
FC accumulator	Signed	32 bits	9	Q23.9
Output probability	Unsigned	8 bits	7	UQ1.7

For a value represented using *W* total bits and *F* fractional bits, the fixed-point integer representation is obtained as Equation 11,

$$x_{q}=x2^{F}$$ (11)

where x denotes the floating-point value, F is the number of fractional bits, and $x_{q\;}$ is the corresponding quantized integer representation. The reconstructed fixed-point value is given by Equation 12,

$$x=x_{q}2^{-F}$$ (12)

The floor operation was consistently used during quantization to match the numerical behavior of the implemented fixed-point model. Signed representations were used for convolution weights, biases, projected feature maps, attention outputs, and accumulators, whereas unsigned representations were used for non-negative input and ReLU feature maps. The specific fixed-point formats and signedness assigned to these quantities are provided in Table 3.

For a signed *W*-bit fixed-point representation with *F* fractional bits, the representable numerical range is given in Equation 13,

$$x_{min}=-2^{W-F-1},\;x_{max}=2^{W-F-1}-2^{-F}$$ (13)

whereas for an unsigned representation is given in Equation 14,

$$x_{min}=0,\;x_{max}=2^{W-F}-2^{-F}$$ (14)

These bounds were used to verify that the selected formats provide sufficient dynamic range for the corresponding intermediate feature maps. In particular, the first two LeakyReLU feature maps were represented using UQ1.7, while the third LeakyReLU output used UQ5.11 because of its larger activation range. The projected feature maps, FAFF output, attention convolution output, attention-gated feature map, and global-average-pooled representation were assigned the signed formats listed in Table 3. The attention sigmoid output and final classification probability were represented using UQ1.7.

For each convolutional operation, multiplication results were accumulated using a signed 40-bit accumulator with 31 fractional bits (Q9.31). The MAC operation can be expressed in Equation 15,

$$A=\sum_{i\;=1}^{N}x_{i}w_{i}+b$$ (15)

where $x_{i}\;$ and $w_{i}\;$ represent the input feature and corresponding weight, respectively, b is the bias, and A is the accumulated result. The accumulation was performed at the higher-precision accumulator format before conversion to the destination feature-map format specified in Table 3. This approach preserves the required binary-point alignment during multiplication and accumulation while limiting quantization to the explicitly defined conversion stages.

To assess the effect of fixed-point representation, the floating-point intermediate activations from all test images were mapped to their corresponding fixed-point formats, and the representable ranges were checked for overflow. No overflow was observed for LeakyReLU1, LeakyReLU2, LeakyReLU3, the three projection branches, FAFF, attention convolution, attention-gated feature map, global average pooling, or the final fully connected activation under the selected formats. Thus, the selected word lengths provide sufficient numerical range for the evaluated test set.

The quantization sensitivity was further evaluated by comparing the floating-point classification probability $P_{i}^{FP}$ with its quantized representation $P_{i}^{Q}$. The absolute probability error for each test sample is defined in Equation 16,

$$e_{i}=|P_{i}^{FP}-P_{i}^{Q}|$$ (16)

The maximum and mean probability errors are calculated from Equations 17, 18,

$$E_{max}=\underset{i\;}{\max}|P_{i}^{FP}-P_{i}^{Q}|$$ (17)

$$E_{mean}=\frac{1}{N}\sum_{i}^{N}\left|P_{i}^{FP}-P_{i}^{Q}\right|$$ (18)

where N is the total number of test images. The obtained maximum and mean probability deviations were 0.00781238 and 0.00151347, respectively. The fixed-point implementation produced the same binary classification decisions as the floating-point implementation for all test images, resulting in 100% prediction agreement. Both the floating-point and fixed-point implementations achieved a classification accuracy of 97.82% on the test set, with identical confusion matrices. Furthermore, no overflow was observed in any of the validated intermediate computational stages, including LeakyReLU1, LeakyReLU2, LeakyReLU3, the three projection branches, FAFF, attention convolution, attention-gated feature map, global average pooling, and the fully connected layer.

These results demonstrate that the selected fixed-point formats provide adequate numerical range for the evaluated intermediate representations and introduce only a small deviation in the final classification probability. The identical classification decisions and accuracy obtained by the floating-point and fixed-point implementations indicate that the selected fixed-point representation preserves the predictive behavior of the CNN on the evaluated test set.

The proposed architecture further employs a reusable time-multiplexed MAC datapath with a sharing factor of 16. This temporal resource-sharing strategy reduces the number of parallel arithmetic resources required for the CNN computation while increasing the number of processing cycles required for complete inference.

## 6 Experimental results

This section shows the experimental analysis of the proposed CNN-FPGA system. The findings justify the classification performance of the proposed model and its successful hardware implementation on the PYNQ-Z2 FPGA platform. The analysis focuses on hardware execution, resource usage, timing performance, and physical output validation.

### 6.1 Experimental setup and dataset description

The experimental evaluation consists of MATLAB-based CNN development and FPGA-oriented implementation and verification. The CNN model was developed and tested in the MATLAB environment to evaluate its classification performance. The trained model was then converted into synthesizable VHDL using MATLAB HDL Coder. The generated HDL implementation was functionally verified using the same preprocessed CT image data used for MATLAB inference, and the HDL classification output was compared with the corresponding MATLAB result. The PYNQ-Z2 FPGA platform was used as the target device for Vivado synthesis, placement, routing, timing analysis, and resource-utilization evaluation. The top-level FPGA wrapper provides the control and LED output interfaces for the implemented CNN design. The experimental configuration is summarized in Table 4.

**Table 4:** Experimental setup and implementation configuration.

Category	Description
Software-Environment	MATLAB
Model	FAA-CNN
Classification	Binary
HDL Generation	HDL Coder
FPGA Design	Xilinx Vivado
FPGA Board & Device	PYNQ-Z2 & Zynq-7020
Hardware-Validation	MATLAB–HDL output comparison

The proposed FAA-CNN was trained using the Adam optimizer with an initial learning rate of 0.001, a mini-batch size of 32, and a maximum of 50 epochs. Binary cross-entropy was employed as the classification loss. The network parameters were randomly initialized, and a dropout rate of 0.5 was used for regularization. A separate validation set was employed during training, while early stopping was not applied. The dataset was divided at the image level into 70% training, 15% validation, and 15% testing subsets. The test set was kept completely independent from model training and parameter tuning and was used only for the final performance evaluation. The final reported FAA-CNN results were obtained from one independent training run.

The dataset utilized in this research was sourced from the publicly accessible Pulmonary Fibrosis Dataset Final repository on Kaggle. The compilation of this study comprises 5,120 CT scans, with 2,560 representing normal instances and 2,560 denoting pulmonary fibrosis instances. Due to the original CT images possessing differing spatial dimensions, all pictures were adjusted to 128 × 128 pixels to ensure a uniform input size for the suggested CNN. The dataset was partitioned into a training set, a validation set, and a testing set at a 70:15:15 ratio, yielding 3,560 images for training, 780 images for validation, and 780 images for testing. The three subsets comprise mutually exclusive image files, with no duplicate image files present in the training, validation, and testing sets. No data enhancement techniques were utilized throughout the model training process. The dataset's distribution by class is illustrated in Table 5.

**Table 5:** Distribution of dataset description.

Dataset Spilt	Training	Validation	Testing	Total
Normal	1,780	390	390	2,560
Pulmonary Fibrosis	1,780	390	390	2,560
Total	3,560	780	780	5,120

As patient-level identifiers were not available in the publicly accessible dataset, patient-level independence could not be explicitly verified; therefore, the possibility of correlations among images from the same patient cannot be completely excluded.

### 6.2 Performance evaluation

The Feature Aware Attention CNN is tested at the software level to perform binary classification with the use of CT images. The model can separate the two target classes with predictive consistency. The CNN architecture has been specified as a lightweight one so that it can fit the FPGA resource constraints and at the same time achieve reasonable classification performance. Figure 5 shows the Sample CT images.

**Figure 5:**
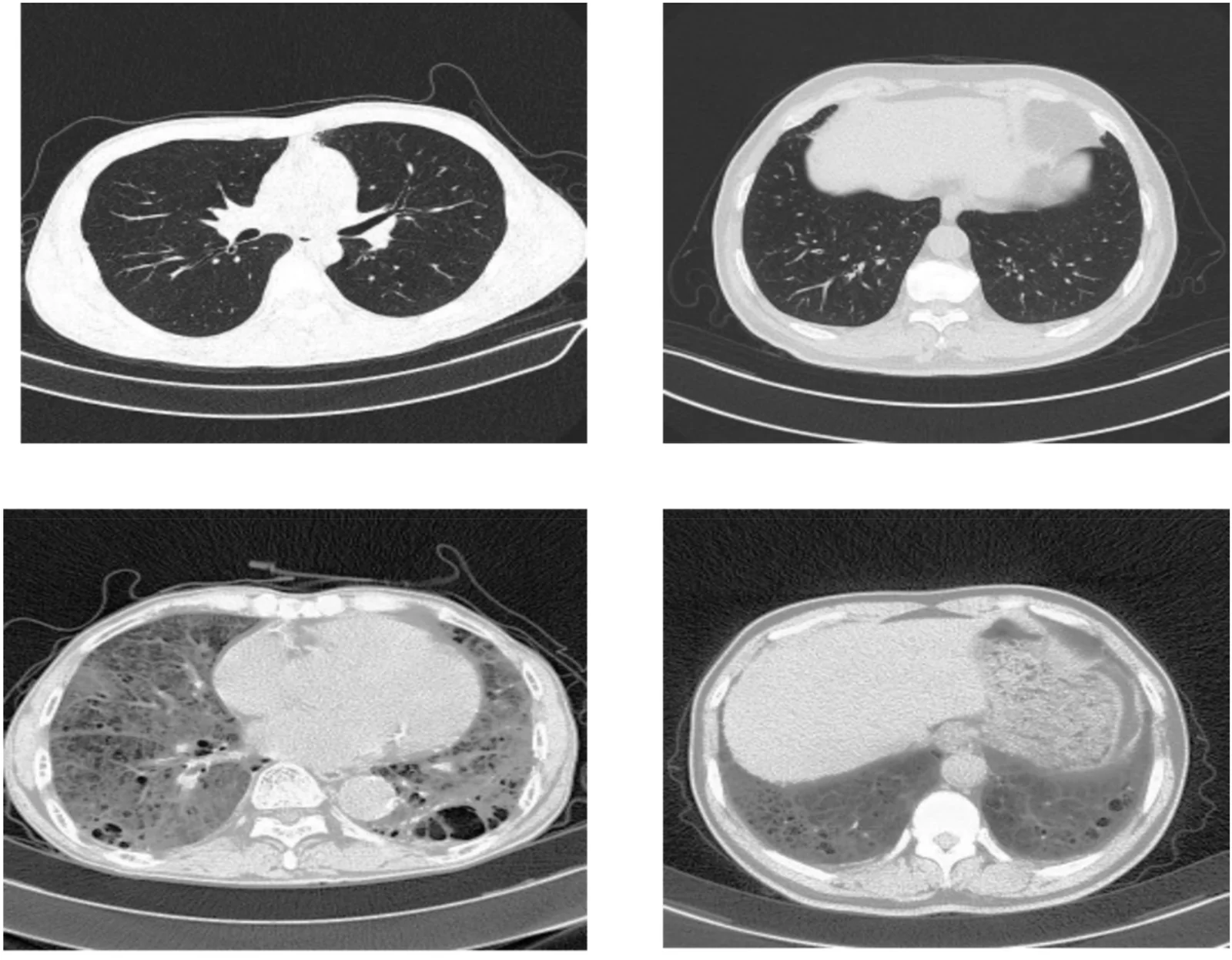
Sample CT images.

#### 6.2.1 Feature visualization analysis

Figures 6a,b show representative feature visualizations for normal and pulmonary fibrosis CT images, respectively. In both cases, the initial convolutional layers mainly extract low-level anatomical boundaries and intensity variation. The deeper convolutional layer captures more discriminative pulmonary structures and texture features. The feature fusion module fuses the information from various convolution layers which generates a complete representation of lung anatomy. The attention mechanism also boosts diagnostically relevant regions, giving more weight to the activation of pulmonary structures and suppressing the irrelevant background regions. Increased activations are found in regions of abnormal lungs for pulmonary fibrosis cases, highlighting the ability of the proposed FAA-CNN to capture the patterns related to pulmonary fibrosis and enhance classification accuracy.

**Figure 6:**
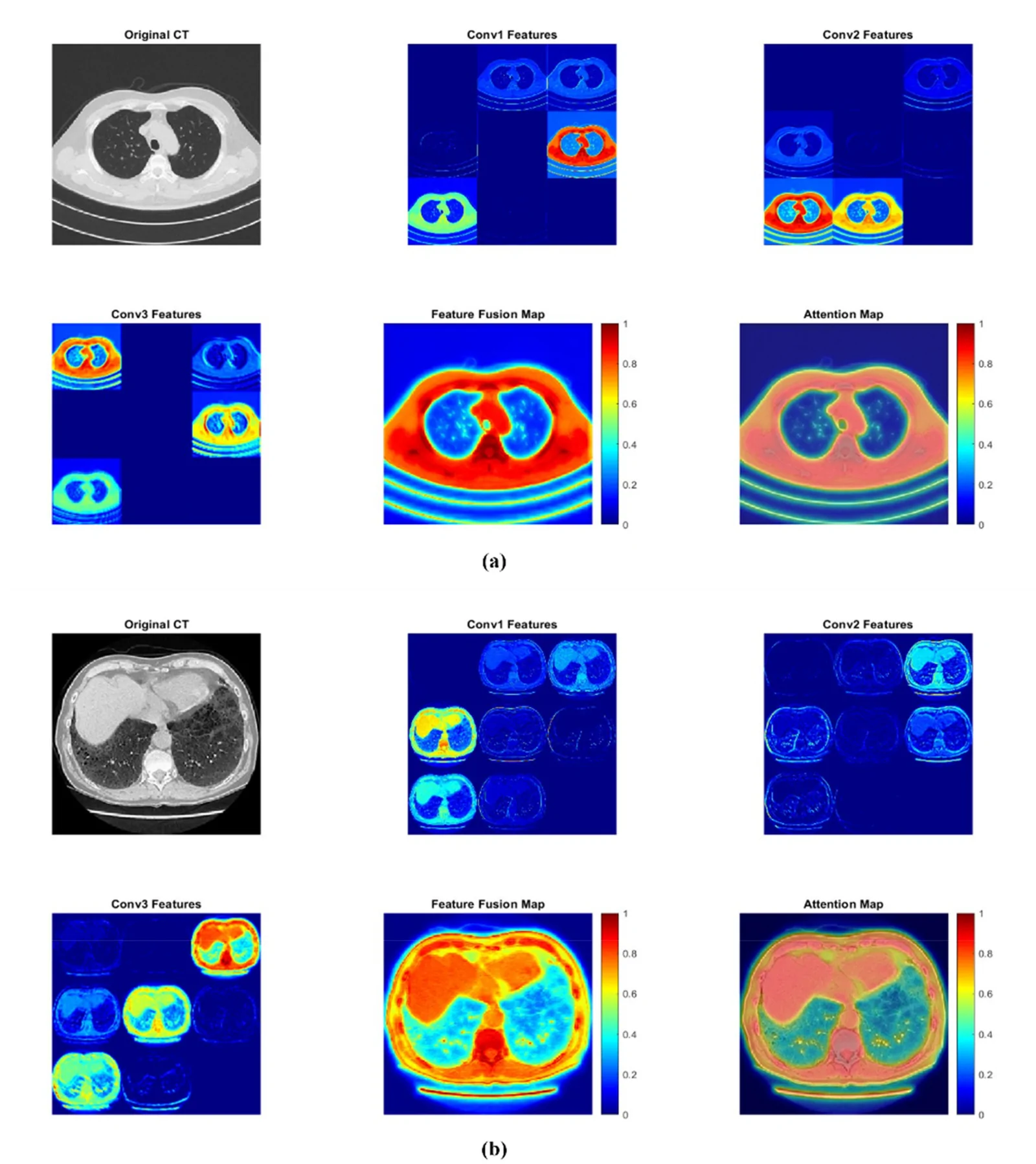
Visualization of intermediate feature representations generated by the proposed FAA-CNN **(a)** normal CT image and **(b)** pulmonary fibrosis CT image.

Figure 6 shows representative intermediate feature-map outputs obtained from selected stages of the proposed FAA-CNN. The activations are visualized as color images to qualitatively illustrate the evolution of learned feature representations across the network. These intermediate feature maps are provided for qualitative inspection of feature extraction and are not interpreted as validated fibrosis-localization maps or as evidence of clinical explainability.

Figure 7 shows the binary classification results obtained from the proposed model. The classification performance of the proposed FAA-CNN is evaluated using standard binary classification metrics to validate model correctness before hardware deployment.

**Figure 7:**
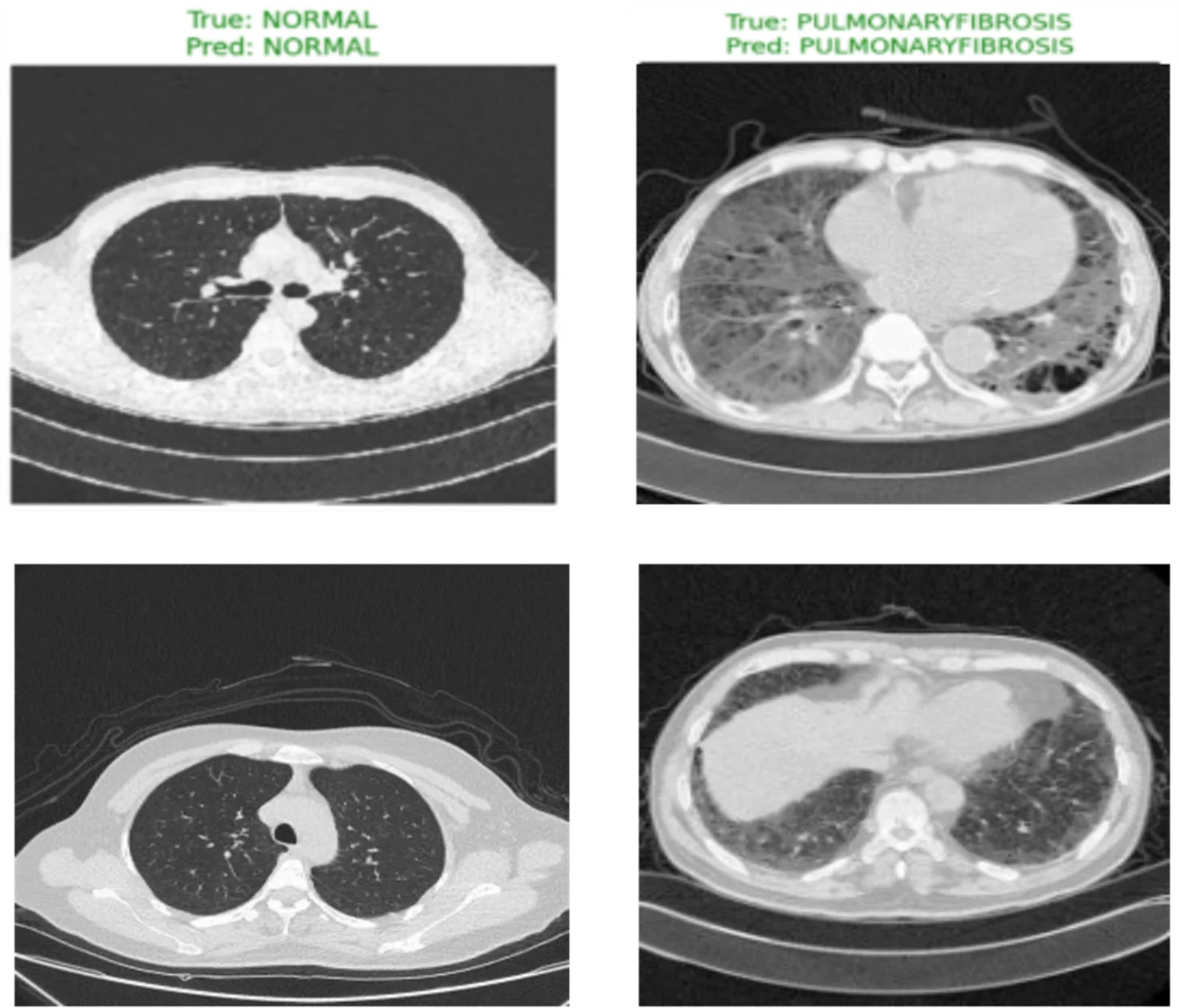
Classification results of the proposed model.

Figure 8 shows the confusion matrix of the proposed model. The performance of the proposed model was estimated on the testing dataset involving 780 images. The experimental results show that 384 normal images and 379 pulmonary fibrosis images were correctly classified. In contrast, misclassifications were observed in 6 normal images and 11 pulmonary fibrosis images.

**Figure 8:**
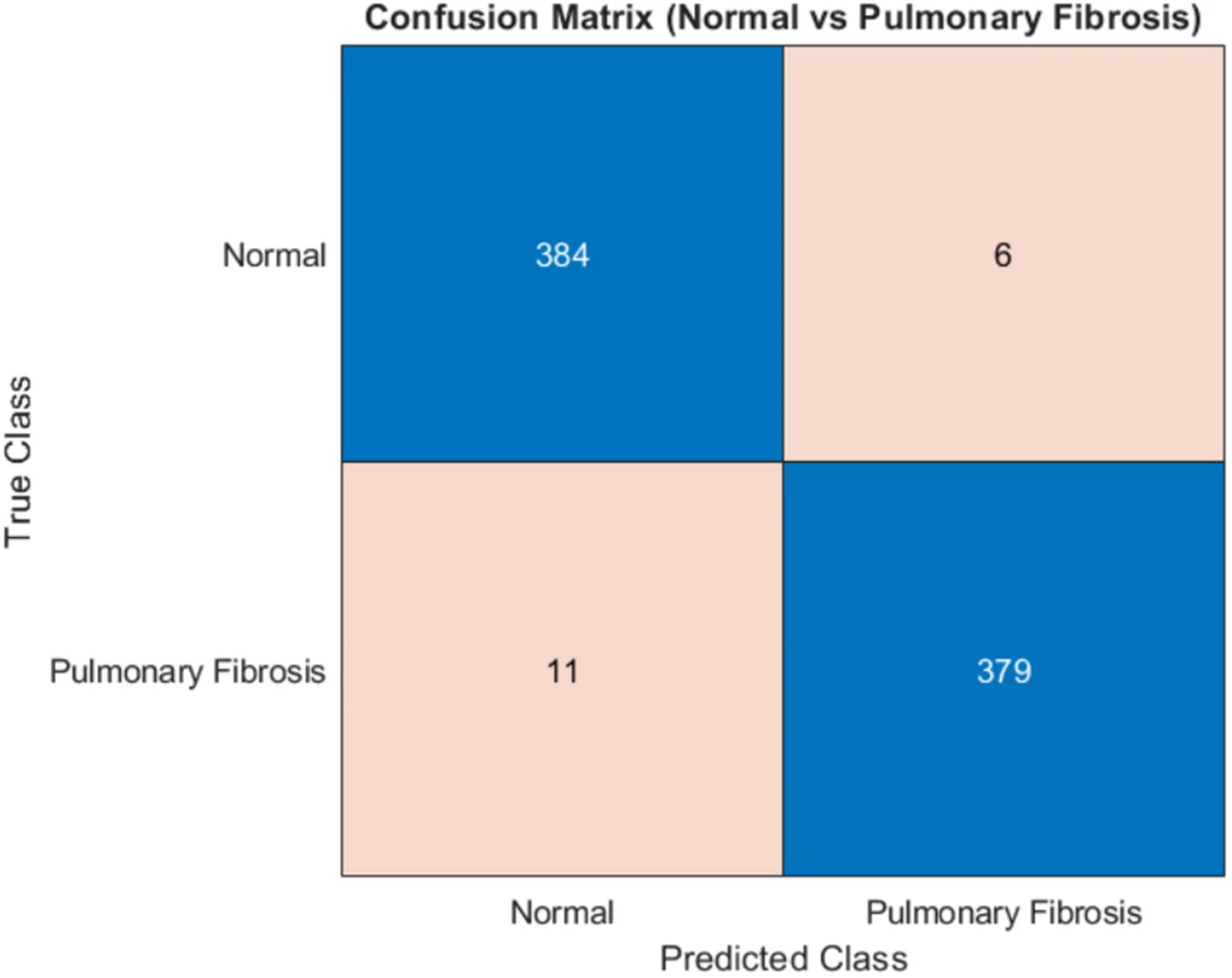
Confusion matrix of the proposed model.

Figure 9 shows the Receiver Operating Characteristic (ROC) curves for the analytical model, baseline CNN, and proposed FAA-CNN. The ROC curve shows the relationship between the True Positive Rate (TPR) and False Positive Rate (FPR) across a range of decision thresholds, with the Area Under the Curve (AUC) representing the area under the entire curve, which indicates the overall classification performance of the model. The ROC analysis validates that the proposed FAA-CNN consistently has a higher true positive rate and lower false positive rate than the analytical and baseline models. The feature fusion and attention mechanisms have significantly boosted the feature representation and highlighted fibrosis-related areas, as evidenced by the improvement in AUC. The results thus confirm the better classification accuracy and stability of the proposed FAA-CNN in the detection of pulmonary fibrosis.

**Figure 9:**
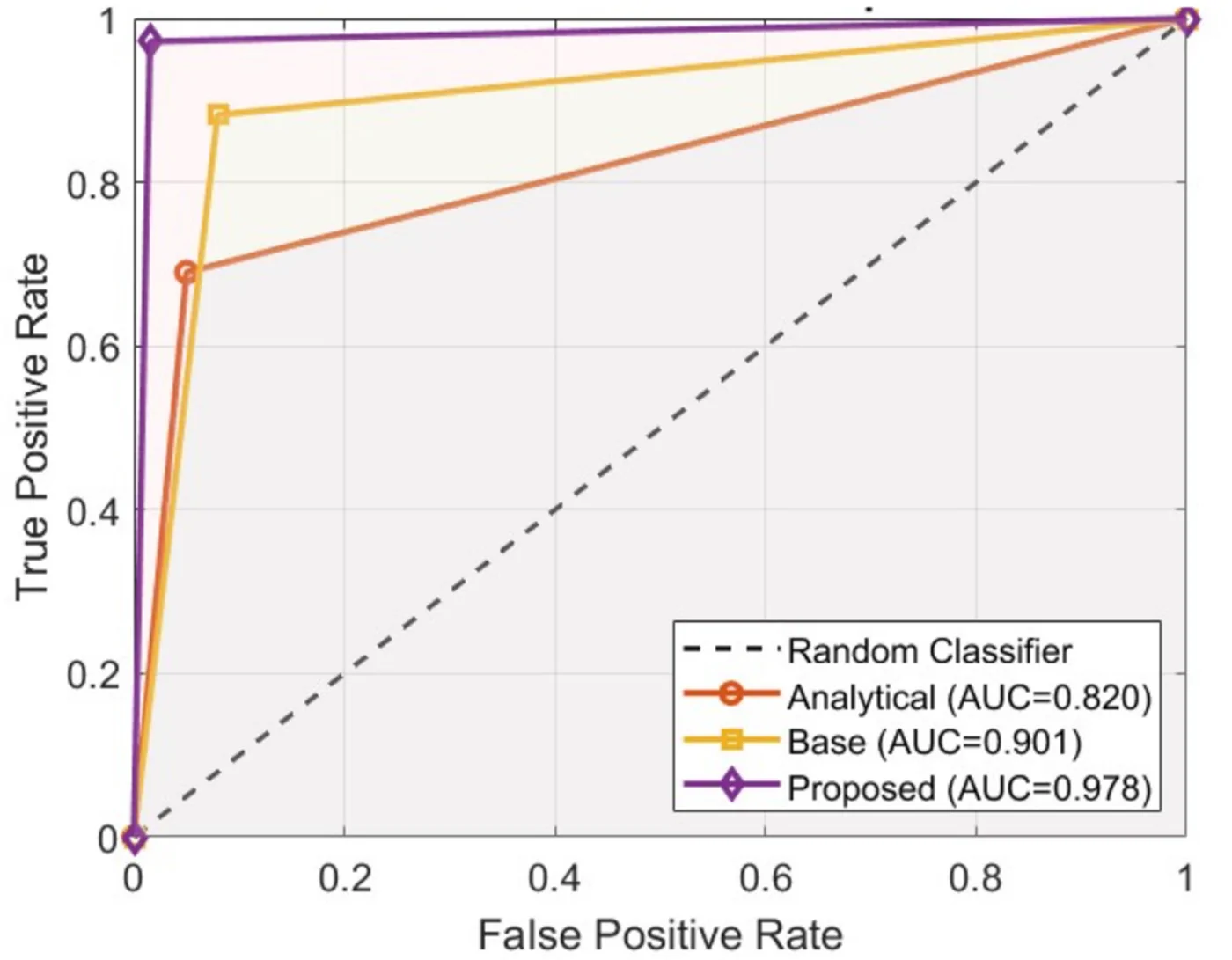
ROC curve with AUC comparison of the analytical model, baseline CNN, and proposed FAA-CNN for pulmonary fibrosis classification.

The baseline model achieves an accuracy of 90%, with low sensitivity and a high false negative rate, indicating limited capability in capturing discriminative CT image features. This observation motivates the design of a task-specific FAA-CNN optimized for embedded FPGA implementation. From the performance comparison table, Sensitivity and false positive rate are particularly important in medical diagnosis, as false positives and false negatives can directly impact clinical decision-making. The analytical model of this threshold-based classifier is applied as a starting point hardware-efficient model and its output is compared against the Base CNN and the proposed CNN to show the superiority of deep learning-based feature extraction.

The outcomes of the three assessed models are encapsulated. The proposed FAA-CNN attained an accuracy of 97.82%, a sensitivity of 97.18%, and a specificity of 98.46%. The 95% confidence intervals for the proposed FAA-CNN are presented together with its related performance measures in Table 6. The performance metrics of the comparison graph are shown in Figure 10. The graph illustrates the performance of the analytical model, the base CNN model, and the proposed CNN model in relation to each other, as measured by various evaluation metrics and green scatter point denotes the proposed model. The enhanced values of the accuracy, sensitivity, and specificity are obtained in the proposed model, and the false positive rates(FPR) and false negative rates(FNR) are significantly lower. This demonstrates the high diagnostic capacity and accuracy of the proposed CNN structure to identify pulmonary fibrosis.

**Table 6:** Performance comparison of classification.

Metric	Analytical model (%)	Base model (%)	Proposed FAA-CNN (%)
Accuracy	82.00	90.13	97.82 (96.54–98.63)
Sensitivity	69.00	88.21	97.18 (95.02–98.42)
Specificity	95.00	92.05	98.46 (96.68–99.29)
False Positive Rate (FPR)	5.00	7.95	1.54
False Negative Rate (FNR)	31.00	11.79	2.82

Values in parentheses indicate 95% confidence intervals calculated using the Wilson binomial method for the proposed FAA-CNN.

**Figure 10:**
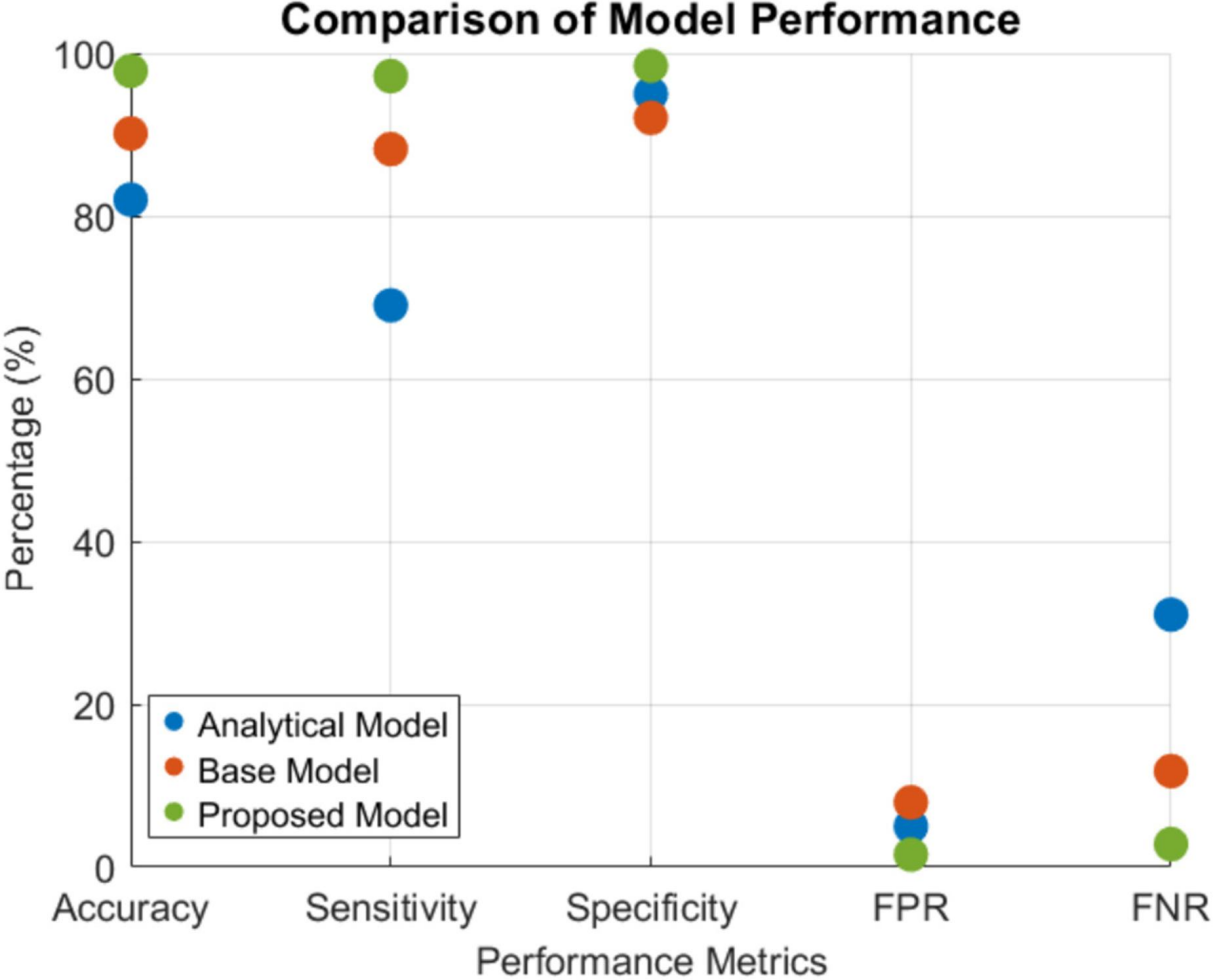
Comparative analysis of all models.

Table 7 illustrates the detailed layer-by-layer architecture of the proposed FAA-CNN, including input/output sizes, convolutional configurations, activation functions, and adjustable parameters. The network encompasses 8,865 adjustable parameters, consisting of 8,680 weights and 185 biases. The low, mid, and high-level feature representations are consolidated into a singular 32-channel dimension before being uniformly integrated by the FAFF module. The attention mechanism then generates a 128 × 128 × 32 attention map for feature-oriented gating.

**Table 7:** Detailed architecture of the proposed FAA-CNN.

Layer no/name	Operation	Kernel/configuration	Input size	Output size	Trainable parameters
1/Input	Image input	—	—	128 × 128 × 1	0
2/Conv1	2-D convolution	3 × 3, 8 filters, stride 1, same padding	128 × 128 × 1	128 × 128 × 8	80
3/ReLU1	ReLU activation	—	128 × 128 × 8	128 × 128 × 8	0
4/Conv2	2-D convolution	3 × 3, 16 filters, stride 1, same padding	128 × 128 × 8	128 × 128 × 16	1,168
5/ReLU2	ReLU activation	—	128 × 128 × 16	128 × 128 × 16	0
6/Conv3	2-D convolution	3 × 3, 32 filters, stride 1, same padding	128 × 128 × 16	128 × 128 × 32	4,640
7/ReLU3	ReLU activation	—	128 × 128 × 32	128 × 128 × 32	0
8/Projection-Low	1 × 1 convolution	1 × 1, 32 filters, 8 → 32 channels	128 × 128 × 8	128 × 128 × 32	288
9/Projection-Mid	1 × 1 convolution	1 × 1, 32 filters, 16 → 32 channels	128 × 128 × 16	128 × 128 × 32	544
10/Projection-High	1 × 1 convolution	1 × 1, 32 filters, 32 → 32 channels	128 × 128 × 32	128 × 128 × 32	1,056
11/FAFF	Element-wise feature fusion	*α*₁ = α₂ = α₃ = 1	3 × (128 × 128 × 32)	128 × 128 × 32	0
12/Attention Conv	1 × 1 convolution	1 × 1, 32 filters	128 × 128 × 32	128 × 128 × 32	1,056
13/Attention Sigmoid	Sigmoid activation	—	128 × 128 × 32	128 × 128 × 32	0
14/Attention Gate	Element-wise multiplication	Feature × attention map	128 × 128 × 32	128 × 128 × 32	0
15/Global Average Pooling	Global Average Pooling	—	128 × 128 × 32	1 × 1 × 32	0
16/Fully Connected	Fully Connected	32 → 1	1 × 1 × 32	1 × 1 × 1	33
17/Sigmoid Output	Sigmoid activation	—	1 × 1 × 1	1 × 1 × 1	0
Total					8,865

Table 8 summarizes the computational complexity of the proposed FAA-CNN for a single 128 × 128 × 1 input image. The convolutional, projection, attention-convolution, and fully connected layers account for the major computational workload, resulting in 141,688,864 MAC operations per inference. Considering one multiplication and one addition per MAC, the corresponding computational requirement is 283,377,728 FLOPs. Element-wise feature fusion, attention gating, pooling, and activation operations are not included in the MAC count. Although the network requires approximately 141.69 million MAC operations per inference, its trainable parameter count is only 8,865, making it parameter-efficient and potentially suitable for resource-constrained hardware when appropriate hardware parallelism and resource-sharing strategies are employed.

**Table 8:** Computational complexity of the proposed FAA-CNN.

Layer	MAC operations
Conv1	1,179,648
Conv2	18,874,368
Conv3	75,497,472
Projection-Low	4,194,304
Projection-Mid	8,388,608
Projection-High	16,777,216
Attention Conv	16,777,216
Fully Connected	32
Total MACs	141,688,864
Total FLOPs	283,377,728

The layer-wise values are consistent in Equation 19,

$$C_{ideal}=\frac{N_{MAC\;\;}\times S}{D}$$ (19)

where $N_{\mathrm{MAC}}\;$ is the number of MAC operations, S is the sharing factor, and D is the number of DSP48E1 slices. Therefore,

$$C_{ideal}=\frac{141,688,864\;\times \;16}{10}\approx 226,702,182\;\mathrm{cycles}.$$

The idealized MAC scheduling calculation results in approximately 226.7 million arithmetic cycles. The measured end-to-end inference latency is 476,856,353 clock cycles (476.9 million cycles), which is higher than the idealized arithmetic requirement because the implemented HDL architecture additionally incurs cycles associated with sequential control, memory access and data movement, address generation, accumulation, intermediate-result handling, pipeline operation, and inter-layer scheduling. Therefore, the 226.7 million cycles represent the idealized MAC-only scheduling requirement, whereas the reported 476.9 million cycles represent the complete end-to-end inference latency of the implemented FPGA architecture. The layer-wise idealized scheduling and the distinction between arithmetic cycles and measured end-to-end latency are detailed in Table 9 and the accompanying calculation.

**Table 9:** Layer-wise MAC scheduling for the FPGA implementation.

Layer	MAC operations	DSP48E1 slices	Sharing factor	Idealized scheduled cycles
Conv1	1,179,648	10	16	1,887,437
Conv2	18,874,368	10	16	30,198,989
Conv3	75,497,472	10	16	120,795,955
Projection-Low	4,194,304	10	16	6,710,886
Projection-Mid	8,388,608	10	16	13,421,773
Projection-High	16,777,216	10	16	26,843,546
Attention Conv	16,777,216	10	16	26,843,546
Fully Connected	32	10	16	51.2
Total	141,688,864	10	16	226,702,182

### 6.3 FPGA resource utilization

The FPGA resource usage is obtained through the Vivado synthesis report. Table 10 provides a summary of the usage of the main resources of the FPGA platform. It is seen that the proposed design does not exceed the limits of the PYNQ-Z2 platform.

**Table 10:** FPGA resource utilization of the proposed model.

Resource	Available	Used	Utilization (%)
LUTs	53,200	5,236	9.84
Flip-Flops	106,400	4,931	4.63
BRAMs	140	106.5	76.07
DSP48E1	220	10	4.55
LUTRAM/Distributed RAM	17,400	0	0
IO	125	38	30.40
BUFG (Clock)	32	1	3.13

The proposed CNN was implemented on a Xilinx Zynq-7000 XC7Z020 FPGA using MATLAB HDL Coder. To reduce hardware resource requirements, the convolutional and fully connected operations were implemented using a sequential, resource-shared architecture rather than fully parallel computation. The convolutional MAC operations are therefore executed over multiple clock cycles using reusable arithmetic resources. The input image and intermediate feature maps are stored in on-chip block RAM, while the implemented arithmetic operations are mapped to FPGA DSP and logic resources. The final design was synthesized, placed, and routed using Vivado 2025.2.

The input frame and intermediate feature maps are stored in on-chip block RAM, while the convolutional MAC operations are mapped to FPGA DSP and logic resources. Resource sharing with a sharing factor of 16 was enabled during HDL generation to reuse arithmetic resources across successive operations. The final post-implementation design uses 5,236 LUTs, 4,931 flip-flops, 106.5 BRAM tiles, and 10 DSP48E1 slices. The design achieves timing closure at 47 MHz, with a WNS of +0.056 ns, TNS of 0 ns, and no setup or hold violations.

### 6.4 Timing performance and latency analysis

The timing performance of the implemented CNN was evaluated using the post-implementation Vivado timing analysis, and the obtained timing and performance results are summarized in Table 11. The final design achieves timing closure at an operating frequency of 47 MHz, corresponding to a clock period of 21.277 ns. The design obtains a worst negative slack (WNS) of +0.056 ns and a worst hold slack (WHS) of +0.047 ns, with no setup or hold violations. The proposed FPGA implementation employs sequential resource sharing with a sharing factor of 16, resulting in approximately 476.9 million cycles per image, corresponding to 10.15 s/image and 0.099 images/s at 47 MHz. This demonstrates a trade-off between reduced hardware resource utilization and increased computational latency.

**Table 11:** Timing and performance summary of the proposed model.

Parameter	Proposed model
Operating Frequency	47 MHz
Clock Period	21.277 ns
Worst Negative Slack (WNS)	+0.056 ns
Worst Hold Slack (WHS)	+0.047 ns
Cycles per Inference*	476,856,353
Inference Latency*	10.15 s
Throughput*	0.099 images/s
MACs per Inference	141.69 M
Operations per Inference	283.38 M
Achieved Computational Rate*	0.028 GOPS

* Cycle-count-derived values based on the sequential execution schedule.

The CNN requires approximately 141.69 million MAC operations for each inference, corresponding to approximately 283.38 million operations when one MAC is regarded as two operations. The resulting computational throughput is approximately 0.028 GOPS. The latency and throughput values are derived from the sequential hardware schedule rather than measured from a physical board-level CT-image inference experiment.

### 6.5 Power and hardware efficiency

The power consumption of the implemented FPGA design was estimated using the Vivado power analysis report at the timing-closed operating frequency of 47 MHz. The performance results are summarized in Table 12. The estimated total on-chip power is 0.139 W, consisting of 0.028 W dynamic power and 0.112 W device static power.

**Table 12:** Power estimation of the proposed model.

Component	Value
Operating Frequency	47 MHz
Total On-Chip Power	0.139 W
Device Static Power	0.112 W
Dynamic Power	0.028 W
Power Analysis	Vectorless Estimate

The reported power reflects the anticipated on-chip power of the FPGA design and does not account for total board-level power usage. The assessment encompasses the FPGA clock, logic, signals, BRAM, DSP, and I/O elements. The deployed CNN utilizes on-chip BRAM and omits external DDR for data access, therefore excluding any power consumption from external DDR memory transfers.

The Vivado power analysis utilizes vectorless activity estimation. Hence, the estimate provided is an approximation rather than a number obtained from empirical measurement. The Vivado report reveals a reduced confidence level stemming from the lack of extensive switching activity data for all internal signals and I/O. Consequently, the referenced 0.139 W is identified as an FPGA on-chip power estimation rather than a genuine board-level power assessment.

### 6.6 FPGA implementation and verification results

The proposed CNN was translated into synthesizable VHDL using MATLAB HDL Coder and subsequently synthesized, placed, and routed for the PYNQ-Z2 FPGA platform using Vivado. The generated VHDL design was functionally verified using the identical preprocessed 128 × 128 CT image data employed for MATLAB inference, and the HDL output was compared with the corresponding MATLAB classification outcome to verify functional consistency. The implemented design was then tested on the physical PYNQ-Z2 board using the sequential 16-bit input interface.

The top-level FPGA wrapper includes the control and LED output interfaces. The binary classification result is mapped to two LED signals, where a probability greater than 0.5 corresponds to pulmonary fibrosis and a probability less than or equal to 0.5 corresponds to the normal class. Figure 11 illustrates the implemented LED output interface for the two classification states. Thus, the HDL functional verification, post-implementation FPGA analysis, and physical board-level CT-image inference are considered as distinct evaluation stages.

**Figure 11:**
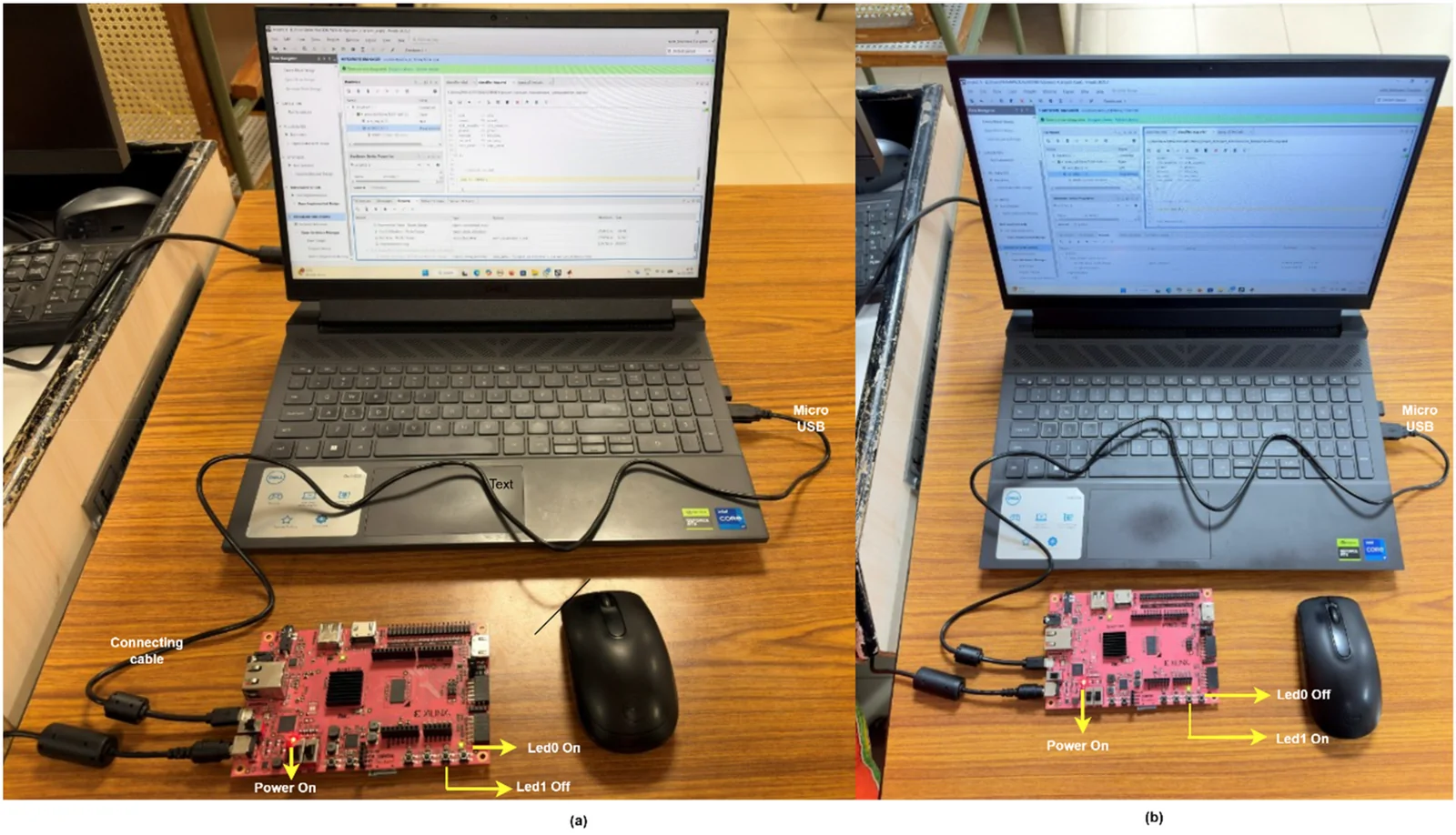
LED output interface of the proposed CNN FPGA implementation for the two binary classification states: **(a)** normal and **(b)** pulmonary fibrosis.

The PYNQ-Z2 was used as the target FPGA platform for hardware synthesis, implementation analysis, and physical board-level testing.

### 6.7 Performance of the ablation study

The experimental results demonstrate that the proposed FAA-CNN can be successfully implemented on FPGA hardware for binary classification. The LED outputs confirm autonomous, software-free hardware inference. The FPGA-based system provides deterministic latency, low power consumption, and suitability for embedded deployment compared to software-only approaches. These results highlight the feasibility of deploying CNN-based medical image classifiers on hardware platforms, bridging the gap between algorithmic research and practical implementation. Ablation study functions are shown in Table 13.

**Table 13:** Ablation study functions.

Model	Fusion	Attention
Proposed	Yes	Yes
Ablation 1	No	Yes
Ablation 2	Yes	No

The results in Table 14 show that the ablation studies indicate that the two mechanisms, feature fusion and attention, play an important role in improving the performance of the pulmonary fibrosis classification. The model without fusion attained an accuracy of 95.26%, while the model without attention achieved 96.03%. The proposed model, which incorporates both fusion and attention modules, achieved an accuracy of 97.82%, as well as sensitivity (97.44%) and specificity (98.21%). Furthermore, the proposed model achieved the minimal false positive rate (1.54%) and false negative rate (2.82%), which represent a higher level of reliability in not only detecting fibrosis but also in detecting normal cases. The results validate the fact that the fusion module enhances the multi-scale feature representation, and the attention mechanism enables the network to focus on disease-relevant regions, leading to an overall improved classification performance.

**Table 14:** Ablation study of FAA-CNN.

Model	Accuracy	Sensitivity	Specificity	Precision	F1 Score	False Positive Rate	False Negative Rate
Without Fusion	0.9526	0.9487	0.9564	0.9561	0.9524	0.0436	0.0513
Without Attention	0.9603	0.9590	0.9615	0.9614	0.9602	0.0385	0.0410
Proposed	0.9782	0.9718	0.9846	0.9844	0.9781	0.0154	0.0282

The fusion of features enhances the performance of classification since it has the ability to combine the multi-scale feature representation, allowing the network to learn both minute texture irregularities and unpolished morphological alterations that accompany the presence of pulmonary fibrosis. The attention mechanism also promotes performance because it prioritizes areas of fibrosis and inhibits insignificant background information. The results of the ablation study also prove the effectiveness of the suggested fusion and attention mechanisms. The non-fusion and non-attention model had lower accuracy due to less feature representation. Fusion of features enhanced the performance by increasing the quality of features whereas the addition of the attention module also increased the accuracy by allowing the network to focus on relevant fibrosis regions. The fusion of both attention and fusion demonstrated the most accurate results of 97%, showing the usefulness of the proposed architecture.

## 7 Discussion

The analysis of the performance shows a clear enhancement in classification accuracy from the analytical model to the proposed CNN-based embedded AI implementation. The analytical classifier had an accuracy of 82% indicates the weakness of threshold-based and mathematically constructed feature decisions. These methods rely on predetermined intensity correlations, which are unable to capture the complicated spatial distributions seen in CT images and give poor classification performance. The CNN model achieved an accuracy of 90%, underscoring the effectiveness of convolutional neural networks in automatically extracting relevant features from medical images. This represents an 8% improvement over the analytical model, highlighting the advantage of learned feature representations compared to manually defined decision criteria. Nonetheless, the baseline CNN demonstrated limitations in discriminating complex features and achieving overall performance.

The proposed FAA-CNN achieved an accuracy of 97.82%, representing improvements of 15.82 percentage points and 7.69 percentage points over the analytical classifier and baseline CNN, respectively.

This improvement can be explained by the optimized network structure, which enhances feature extraction and classification capability. The classification performance and FPGA hardware efficiency are evaluated as separate aspects of the proposed system. From the hardware perspective, the FPGA implementation was evaluated using resource utilization, timing, latency, throughput, and power estimation. The final design uses 5,236 LUTs (9.84%), 4,931 flip-flops (4.63%), 106.5 BRAM tiles (76.07%), and 10 DSP48E1 slices (4.55%). The design achieves timing closure at 47 MHz, with a WNS of +0.056 ns and a WHS of +0.047 ns. Based on the sequential execution schedule, the cycle-count-derived inference latency is approximately 10.15 s per image, corresponding to approximately 0.099 images/s. The estimated total on-chip power is 0.139 W, comprising 0.028 W dynamic power and 0.112 W device static power.

Overall, the results demonstrate the feasibility of the proposed CNN-based pulmonary fibrosis classification framework on a resource-constrained FPGA platform.

### 7.1 Limitations and generalizability

The present study has several limitations that should be considered when interpreting the results. First, although the dataset contains 5,120 CT images with an equal number of Normal and Pulmonary Fibrosis images, its size and composition may not fully represent the variability encountered in broader clinical populations. Second, the dataset was divided at the image-file level into training, validation, and testing subsets. While identical image files were not shared across the subsets, patient-level identifiers were not available; therefore, patient-level independence between the dataset partitions could not be independently verified. A potential risk of patient-level data leakage cannot consequently be completely excluded, which may result in optimistic performance estimates. Third, although the dataset is balanced at the image-count level, this does not necessarily ensure balanced patient-level representation or demographic and clinical diversity. Finally, the proposed framework has not yet been externally validated on independent multi-center datasets or evaluated across different scanners, acquisition protocols, and patient populations. Future studies should therefore incorporate patient-level data splitting, larger and more diverse multi-center datasets, external validation, and prospective evaluation to establish the generalizability and clinical utility of the proposed approach.

Accordingly, the reported 97.82% accuracy should be interpreted cautiously, as patient-level independence could not be established and potential patient-level data leakage cannot be completely excluded. In addition, the absence of external validation and the use of a single public dataset limit the generalizability of the results to independent clinical populations and imaging settings. Therefore, the proposed framework should be considered a research-stage FPGA-based classification system rather than a clinically validated diagnostic system.

Furthermore, classification performance and FPGA hardware efficiency represent distinct evaluation aspects. Accuracy, sensitivity, specificity, precision, and F1-score assess the predictive performance of the CNN, whereas hardware efficiency is evaluated using implementation metrics such as resource utilization, inference latency, throughput, computational rate, and power consumption.

## 8 Conclusion

This paper presented an AI-based FPGA-oriented implementation of a custom convolutional neural network for binary CT image classification. The proposed framework integrates MATLAB-based CNN development with HDL generation using MATLAB HDL Coder and FPGA implementation targeting the PYNQ-Z2 platform. The experimental findings indicate that the proposed CNN attained a classification accuracy of **97%**, surpassing the analytical model accuracy of 82% and the baseline CNN model accuracy of 90%. The enhanced performance demonstrates the effectiveness of the redesigned CNN architecture for feature extraction and pulmonary fibrosis classification. Feature fusion enhances the representation of complementary features, while the attention mechanism emphasizes relevant feature responses and suppresses less informative regions.

From the FPGA implementation perspective, the proposed sequential resource-shared architecture uses 5,236 LUTs (9.84%), 4,931 flip-flops (4.63%), 106.5 BRAM tiles (76.07%), and 10 DSP48E1 slices (4.55%). The implementation achieves timing closure at 47 MHz, with a WNS of +0.056 ns and WHS of +0.047 ns. Based on the sequential execution schedule, the cycle-count-derived inference latency is approximately 10.15 s per image, corresponding to approximately 0.099 images/s. The Vivado power analysis estimates 0.139 W total on-chip power, comprising 0.028 W dynamic power and 0.112 W device static power. These results demonstrate the feasibility of implementing the proposed CNN on a resource-constrained FPGA platform while maintaining a manageable hardware resource requirement.

The CNN functionality was verified through HDL simulation by comparing the HDL classification output with the corresponding MATLAB result. The FPGA implementation also provides LED-based output mapping for the two classification states. However, further physical board-level inference experiments with direct CT-image acquisition are required to establish practical deployment performance.

Future research will extend the proposed architecture to multi-class classification, support larger and more complex CNN models, and incorporate medical image acquisition interfaces for autonomous embedded operation. Further optimization of the sequential architecture will also be investigated to reduce inference latency and improve throughput. The exploration of advanced architectures, including CNN–Transformer and CNN–Mamba models, represents an additional direction for future work.

## Data Availability

The original contributions presented in the study are included in the article/Supplementary Material, further inquiries can be directed to the corresponding author/s.
